# The situation during the COVID-19 pandemic: A snapshot in Germany

**DOI:** 10.1371/journal.pone.0245719

**Published:** 2021-02-12

**Authors:** Niclas Kuper, Nick Modersitzki, Le Vy Phan, John Rauthmann

**Affiliations:** Bielefeld University, Bielefeld, Germany; Aalborg University, DENMARK

## Abstract

During government-implemented restrictions in the wake of the COVID-19 pandemic, people’s everyday lives changed profoundly. However, there is to date little research chronicling how people perceived their changed everyday lives and which consequences this had. In a two-wave study, we examined the psychological characteristics of people’s situations and their correlates during shutdown in a large German sample (*N*_T1_ = 1,353; *N*_T2_ = 446). First, we compared characteristics during government-issued restrictions with retrospective accounts from before and with a follow-up assessment 6 to 7 months later when many restrictions had been lifted. We found that mean levels were lower and variances were higher for most characteristics during the shutdown. Second, the experience of certain situation characteristics was associated in meaningful and theoretically expected ways with people’s traits, appraisals of the COVID-19 crisis, and subjective well-being. Lastly, situation characteristics often substantially explained the associations of traits with appraisals and well-being. Our findings highlight the importance of considering perceived situations as these contribute to people’s functioning during crises.

## Introduction

The COVID-19 pandemic will likely be remembered as one of the most incisive and consequential events of the 21^st^ century with profound effects on individuals, companies, and countries around the world for years to come. While SARS-CoV-19 poses a significant health threat, it is also the different restrictions by governments (e.g., lockdowns, shutdowns, restrictions of travel, etc.) that have disrupted and changed individuals’ daily lives and pose additional kinds of psychological threats (e.g., isolation, loneliness, anxiety, depression; see [1]). This has led to a flurry of studies and even consortia (e.g., [2–4]) examining the psychological, social, and societal consequences of the pandemic and government-implemented restrictions to combat it. Many psychological studies have focused on individual or dispositional aspects such as personality traits, attitudes, and mental health. For instance, research has indicated that personality traits are associated with well-being during and appraisals of the COVID-19 crisis (e.g., [5–8]).

However, to date people’s experiences of their subjective life-space during the acute phase of the COVID-19 pandemic remain poorly understood (cf. [9]). To truly understand physical and mental functioning during the pandemic, it is vital to have information on both person- and environment-related aspects as well as their relations [10]. This study seeks to fill this gap by assessing subjective situation perceptions in a large German sample during government-implemented restrictions. In doing so, we examine not only how typical situations during the pandemic were experienced, but also how those experiences relate to personality traits, well-being, and appraisals of the COVID-19 crisis.

## Background

It is a truism that person variables (e.g., personality traits and states) and environment variables (e.g., situations and socio-ecological niches) together cast a fuller picture of human functioning [10]. This should also be the case in the context of the COVID-19 pandemic and its consequences. For example, the role of cultural variation in COVID-19 has been emphasized and preliminarily examined [11–13], suggesting that macroscopic cultural aspects–environmental forces–may be relevant for population health during a pandemic. Further, it has been argued that people’s assessments of their risk of contracting COVID-19 may depend on both dispositional and situational attributions [14], suggesting that people’s situations may need to be taken into account in COVID-19 related studies. Another important question concerns changes in psychological variables and functioning. For example, Sutin and colleagues [15] found in a pre-post-test design for a large US sample that self-reported Big Five traits changed across the acute phase of the COVID-19 outbreak. As a mechanism explaining such rapid trait change, the authors surmised that “the broader social environment may be modifying both how individuals see themselves … and the meaning of specific items to how they measure a trait …” (p. 15). Thus, people’s socio-ecological environments and the experiences of their situations may be a driving factor of trait change.

Research has shown that personality traits are associated in meaningful ways with people’s situation experiences [16–18], and those situation experiences predict cognitive-affective outcomes [19]. With the advent of novel approaches to conceptualizing, measuring, and taxonomizing situational information in a psychologically meaningful way [20–24], it has become possible to examine people’s situation experiences and person-situation relations in a fine-grained manner [10, 25]. This work makes use of a novel taxonomy of psychological situation characteristics that capture the interpretations and meanings of situations: The Situational Eight DIAMONDS [20]. The DIAMONDS consist of the following dimensions: Duty (e.g., a job needs to be done), Intellect (e.g., situation contains intellectual stimuli), Adversity (e.g., one is being criticized), Mating (e.g., physical attractiveness is relevant), pOsitivity (e.g., the situation is pleasant), Negativity (e.g., the situation could elicit stress), Deception (e.g., it is possible to deceive someone), and Sociality (e.g., close personal relationships are important or could develop). These dimensions can be used to examine people’s everyday situations, can be captured with several validated measures, and predict reports of affect and behavior (for a summary, see [26]). For these reasons, we use this taxonomy in the current study to better understand people’s lives during the acute phase of the COVID-19 pandemic and government-issued restrictions.

### The current study

The current work provides a window into the characteristics of people’s lives during the COVID-19 pandemic in Germany. In doing so, we (1) compare people’s situation experiences pertaining to the time before (retrospectively assessed) versus during COVID-19 restrictions versus at a follow-up when many restrictions had been lifted, (2) replicate and extend previous work on associations between traits and situation characteristics [16–18, 25], and (3) examine the pathways from traits to important outcomes via situation characteristics. Given our focus on subjectively rated situation characteristics, our findings pertain to situation *experience* (i.e., we do not disentangle actual situation contact from situation construal [17]). Our study takes advantage of the unique context that the COVID-19 pandemic provides for understanding person-environment relations [10] and simultaneously furthers our understanding of people’s everyday lives during this crisis. We formulated three specific research questions tackling person-environment relations during COVID-19.

First, *how did people experience their typical daily situations during COVID-19 restrictions compared to before and after COVID-19 restrictions*? Participants described the characteristics of their typical situations during COVID-19 restrictions along the DIAMONDS dimensions. Additionally, they provided retrospective accounts of their typical situation characteristics before the government-issued COVID-19 restrictions. Since these retrospective reports did not actually come from the time before the restrictions, they have to be interpreted with some caution. The use of retrospective pretest designs is advocated in some research areas but is not without criticism [27–29]. Moreover, these designs may primarily measure subjective perceptions of change rather than actual change [27]. To address this limitation, we conducted a follow-up survey at a timepoint when many COVID-19 restrictions had been lifted in Germany. This allowed us to compare situation ratings pertaining to before (retrospectively assessed) versus during the restrictions versus after many restrictions had been lifted. We quantified mean differences as well as their correlations (rank-order consistencies). We expected at least modest correlations (in line with previous research: e.g., [25]) but also some mean-level differences such that some situation characteristics should be decreased during the crisis due to restrictions (e.g., Sociality: less face-to-face interactions). An interesting ancillary question is to what extent individual differences in the perceptions of the situation characteristics (i.e., variances) were larger or smaller during the restrictions. Although we expected in both cases sizable individual differences (in line with previous research: e.g., [30]), they could be elevated for during- *or* before / follow-up ratings. For example, situations during the restrictions may decrease degrees of freedom and thus afford similar perceptions across persons (see strong situation hypothesis: [31]), suggesting smaller individual differences (variances) for during-related ratings. In contrast, situations may become more heterogeneous during COVID-19 with opposite trajectories for different people (e.g., some people rarely encountered other people if they lived alone, while others were always around family or roommates during the crisis). One may also argue that our highly structured daily lives restrict meaningful variance, and lockdowns/shutdowns provide unstructured situations (often at home) which may coincide with higher levels of individual differences (i.e., larger between-person variances) in during-related ratings. Thus, it is an interesting question at which timepoint more individual differences are observed.

Second, *what are the correlates of people’s situation experiences during the COVID-19 restrictions*? Specifically, we were interested in correlations between DIAMONDS experiences with people’s traits (Big Five, Honesty-Humility, Dark Triad), general negative appraisal of the COVID-19 crisis, and subjective well-being during the COVID-19 restrictions. For personality traits, we focused on individual differences in the Big Five traits, each consisting of three facets [32]: Extraversion (sociability, assertiveness, energy level), Agreeableness (compassion, respectfulness, trust), Conscientiousness (organization, productivity, responsibility), Neuroticism (anxiety, depression, emotional volatility), and Openness (intellectual curiosity, aesthetic sensitivity, creative imagination). Moreover, we included Honesty-Humility (sincerity, fairness, greed-avoidance, modesty [33]) and the Dark Triad traits Narcissism, Machiavellianism, and Psychopathy [34] given their potential relevance for different outcomes during the COVID-19 crisis [7–9]. Previous research has shown that personality traits are associated in meaningful ways with situation experiences [16–18, 25, 30, 35]. For an overview of hypothesized associations between personality traits and situation characteristics, see Table 1. These predictions were based on theoretical considerations and conceptual relationships between situation characteristics and traits. For instance, Duty and Conscientiousness, Intellect and Openness, Negativity and Neuroticism, as well as Sociality and Extraversion are conceptually closely related. Our hypotheses were further based on previous work by Rauthmann and colleagues [25]. Their predictions were extended in the following ways: All Dark Triad traits were predicted to be associated with Adversity, Deception, and Mating given their antagonistic core, relationship to manipulation, and association with mating strategies [36, 37]. For conceptual consistency and due to its inverse relation with Dark Triad Traits [38], Honesty-Humility was additionally predicted to be associated with Adversity. Finally, Narcissism was predicted to be associated with Sociality given the centrality of social interaction dynamics to Narcissism [39]. With respect to well-being and negative appraisals of the COVID-19 crisis, we expected experiences of more adverse, negative, and deceptive as well as less positive and less social situations to coincide with a more negative appraisal of the COVID-19 crisis and the restrictions, as well as with less subjective well-being during the restrictions.

**Table 1:** Theoretically expected links between personality traits and situation characteristics.

	Dut	Int	Adv	Mat	Pos	Neg	Dec	Soc
E				+	+	-		+
A			-				-	+
C	+							
N			+		-	+	+	
O		+						
H			-				-	
Narc			+	+			+	+
Mach			+	+			+	
Psyc			+	+			+	

+ indicates that a positive correlation between the respective situation characteristic and personality trait is theoretically expected,—that a negative correlation is expected. Dut = Duty, Int = Intellect, Adv = Adversity, Mat = Mating, Pos = pOsitivity, Neg = Negativity, Dec = Deception, Soc = Sociality, E = Extraversion, A = Agreeableness, C = Conscientiousness, N = Neuroticism, O = Openness, H = Honesty-Humility, Narc = Narcissism, Mach = Machiavellianism, Psyc = Psychopathy.

Third, *to what extent can situation experiences explain the relations between traits with general negative appraisal of the COVID-19 crisis and subjective well-being*, *respectively*? This question concerns the nexus between traits, situation experiences, and important psychological outcomes (appraisals, well-being) during COVID-19 restrictions. From previous research, we know that personality traits are related to appraisals and well-being in general (e.g., [40, 41]) and during the COVID-19 pandemic [5, 7, 8]. However, it is an open question to what extent experienced situation characteristics explain those associations and which situation perception dimensions are particularly important. Personality traits color how we perceive our worlds [10, 30, 42–44] and both traits and situations govern our mental functioning and well-being. Thus, situation experiences may be one potential explaining mechanism for associations between personality traits and certain outcomes. This suggests paths of traits (independent variables) to appraisal or well-being (dependent variables) via situation experiences (mediators). Thus, our question can be addressed by mediation analyses. Mediation analyses are causal process models that work best with longitudinal and/or experimental data, and they should be applied with caution to cross-sectional data such as ours (pertaining to the timepoint during COVID-19 restrictions) [45, 46]. Although we make use of them as a data-analytical tool to examine this nexus, we do not subscribe to a causal interpretation of the relations (see also *Limitations and Outlook*).

## Method

### Participants and procedure

This research meets all applicable standards for the ethics of experimentation and research integrity, in line with the declaration of Helsinki. Informed consent was obtained from participants and all data was anonymized. No additional local IRB approval was obtained since this is not customary for every questionnaire-based study at German universities. The first assessment (T1) of the present study was conducted between April 3 and April 19, 2020. During this time period, nationwide restrictions (e.g., restrictions on physical social contact and shutdowns of most businesses, facilities, schools, and universities) were in place in Germany [47]. We recruited participants via social media advertisements, university mailing lists, and word-of-mouth. The study took approximately 30 minutes to complete and included information on participants’ self-reported personality traits, perception of their situations before and during COVID-19 restrictions, and demographic variables. As an incentive, participants could receive automatically generated personality feedback. Psychology students received course credit. For more details on the study, see [7] and osf.io/buvp2. We invited 995 participants who agreed to be contacted again to take a follow-up survey. This second assessment (T2) of the present study was conducted between October 20 and November 1, 2020. It included (among other measures not relevant to the present study) measures of participants’ situation characteristics, well-being, and general negative appraisal of the COVID-19 crisis. At this point in time, many of the strict nationwide restrictions had long been lifted [48]. In particular, the shutdown was no longer in place (e.g., businesses, facilities, and schools were open) and there were fewer restrictions on social contact, although stricter rules applied locally in areas where case numbers were surging. Throughout this time period, case numbers in Germany kept increasing substantially. As a consequence, a second but less restrictive “lockdown light” started on November 2, the day after our follow-up assessment had been completed.

For the first assessment, our initial sample consisted of *N* = 1,547 participants who provided data for our questionnaire measures of interest. Exclusion criteria were: age below 18 (*n* = 4); not residing in Germany during the lockdown (*n* = 4); careless responding as indicated by identical responses throughout questionnaires (*n* = 5); duplicate responses as indicated by participant ID codes and email addresses (*n* = 1). Given that we sought to control for age and gender in robustness analyses, we further excluded participants with incomplete age or gender information at both timepoints (*n* = 174) and participants who did not identify as male or female (*n* = 6). Our final sample consisted of *N* = 1,353 participants (58.68% female) with a mean age of *M* = 44.46 years (*SD* = 14.30, range = 18–88). Participants came from all 16 German states, had varying (highest) educational qualifications (*n* = 1,258 responded; e.g., 23.69% had a Master’s degree; 19.40% had completed vocational training; 15.26% had finished intermediate secondary school), and different occupational backgrounds (*n* = 1,342 responded; e.g., 52.91% were employees; 12.37% were college students; 12.00% were retirees). Out of these *N* = 1,353 participants, *N* = 446 had complete data for the follow-up assessment (60.09% female; *M* = 47.08 years old, S*D* = 14.50, range = 19–79; from all 16 states; education: *n* = 413; e.g., 30.27% Master’s degree; 18.16% vocational training; 13.56% intermediate secondary school; occupation: *n* = 445; e.g., 51.01% employees; 10.56% college students; 15.73% retirees).

### Measures

#### Personality traits

Participants filled out the German adaptation of the 30-item Big Five Inventory-2 (BFI-2-S; [49]) using a five-point Likert-type scale (1 = *disagree strongly*; 5 = *agree strongly*). For the assessment of Honesty-Humility, we selected the six HEXACO-60 [33] items with the highest factor loadings in the German translation [50]. This led to the inclusion of two items each for the facets sincerity and fairness, and one item each for the facets greed avoidance and modesty. In the present study, we assessed the Big Five traits and Honesty-Humility rather than the HEXACO traits. It is an actively debated issue in personality psychology whether the Big Five or HEXACO taxonomy is to be preferred [51]. Our pragmatic approach facilitates both the inclusion of additional relevant personality variance in Honesty-Humility and the comparison with previous work on the originally-rotated Big Five [52]. Lastly, Dark Triad traits were assessed using the Naughty Nine scales [53], with a nine-point Likert-type scale (1 = *does not apply at all*; 9 = *fully applies*). Our personality trait measures largely had acceptable internal consistencies (E: ω = .73; A: ω = .69; C: ω = .76; N: ω = .82; O: ω = .72; H: ω = .54; Narc: ω = .84; Mach: ω = .76; Psyc: ω = .59; for descriptives see S1 Table), with some exceptions that are likely attributable to the relatively small number of items.

#### Situation characteristics

Situation experiences were assessed using adaptations of the German version of the S8* [54]. For each DIAMONDS factor, we selected the two items with the highest factor loadings [55]. Note that for Sociality, we decided not to include the second highest loading item (i.e., *Others show many communicative signals*) but the remaining third item (i.e., *Close personal relationships are important or can develop*) which we felt would be easier to understand. Item wordings were adjusted so that they did not refer to a single situation but to daily life in general (i.e., *In my daily life…* or *My daily situations…*). Participants responded on a five-point Likert-type frequency scale (1 = *(almost) never*; 5 = *(almost) always*). When retrospectively reporting on their situations before COVID-19 restrictions, participants were instructed to recall what their daily lives were like in February 2020. For typical situations during COVID-19, participants were asked to think about the time since March 23, 2020, the day after extensive nationwide restrictions had been imposed [47]. For the follow-up assessment, participants were asked to report on their typical situations during the last four weeks before the assessment. The internal consistencies of our situation characteristic scores (computed as Spearman-Brown corrected inter-item correlations [56]) were acceptable to good (with the exception of Mating), considering the fact that only two items were used for each dimension (Table 2).

**Table 2:** Descriptive statistics of situation characteristics.

	*M* before	*M* during	*M* difference	*M* difference *d*_z_	Consistency *r*	*SD* before	*SD* during	Variance Ratio	IC before	IC during
Dut	4.34	3.97	*t* = -15.44, *p* < .001	*d*_z_ = -0.42 [-0.47, -0.37]	0.56	0.79	1.02	1.66 [1.50, 1.85]	0.85	0.85
Int	3.55	3.11	*t* = -17.12, *p* < .001	*d*_z_ = -0.47 [-0.51, -0.42]	0.58	0.96	1.05	1.20 [1.12, 1.29]	0.83	0.83
Adv	2.09	2.01	*t* = -4.22, *p* < .001	*d*_z_ = -0.11 [-0.17, -0.06]	0.61	0.79	0.85	1.17 [1.09, 1.27]	0.71	0.76
Mat	2.41	2.10	*t* = -14.95, *p* < .001	*d*_z_ = -0.41 [-0.46, -0.36]	0.61	0.88	0.84	0.92 [0.85, 0.99]	0.39	0.35
Pos	3.68	3.25	*t* = -16.93, *p* < .001	*d*_z_ = -0.46 [-0.51, -0.41]	0.40	0.76	0.91	1.42 [1.30, 1.55]	0.81	0.83
Neg	3.24	3.09	*t* = -5.47, *p* < .001	*d*_z_ = -0.15 [-0.20, -0.10]	0.48	0.92	1.00	1.19 [1.10, 1.28]	0.77	0.79
Dec	2.29	2.21	*t* = -3.60, *p* < .001	*d*_z_ = -0.10 [-0.15, -0.05]	0.68	0.97	0.96	0.99 [0.92, 1.06]	0.77	0.76
Soc	4.03	3.62	*t* = -16.27, *p* < .001	*d*_z_ = -0.44 [-0.49, -0.39]	0.50	0.83	1.01	1.50 [1.38, 1.63]	0.64	0.65
SWB	70.79	56.67	*t* = -22.99, *p* < .001	*d*_z_ = -0.62 [-0.68, -0.57]	0.34	17.47	21.37	1.50 [1.36, 1.65]	0.85	0.86
GNA		2.59					0.66			0.78
	*M* T2	*M* during	*M* difference	*M* difference *d*_z_	Consistency *r*	*SD* T2	*SD* during	Variance Ratio	IC T2	IC during
Dut	4.20	3.97	*t* = -5.07, *p* < .001	*d*_z_ = -0.24 [-0.32, -0.15]	0.45	0.83	0.99	1.43 [1.19, 1.73]	0.80	0.85
Int	3.42	3.20	*t* = -5.08, *p* < .001	*d*_z_ = -0.24 [-0.33, -0.15]	0.59	0.96	1.07	1.24 [1.09, 1.42]	0.78	0.84
Adv	2.11	2.00	*t* = -2.68, *p* = .008	*d*_z_ = -0.13 [-0.22, -0.03]	0.39	0.74	0.84	1.30 [1.08, 1.56]	0.65	0.75
Mat	2.02	2.02	*t* = 0.03, *p* = .978	*d*_z_ = 0.00 [-0.09, 0.10]	0.41	0.76	0.83	1.19 [1.03, 1.39]	0.40	0.34
Pos	3.35	3.25	*t* = -2.80, *p* = .005	*d*_z_ = -0.13 [-0.22, -0.04]	0.57	0.77	0.92	1.44 [1.24, 1.67]	0.73	0.85
Neg	3.27	3.07	*t* = -4.08, *p* < .001	*d*_z_ = -0.19 [-0.28, -0.10]	0.44	0.90	1.01	1.26 [1.09, 1.46]	0.74	0.81
Dec	2.38	2.27	*t* = -2.30, *p* = .022	*d*_z_ = -0.11 [-0.20, -0.02]	0.46	0.98	0.97	0.98 [0.84, 1.16]	0.78	0.78
Soc	3.86	3.63	*t* = -4.82, *p* < .001	*d*_z_ = -0.23 [-0.32, -0.14]	0.42	0.83	1.00	1.46 [1.23, 1.71]	0.51	0.67
SWB	62.01	58.02	*t* = -4.23, *p* < .001	*d*_z_ = -0.20 [-0.29, -0.11]	0.55	20.78	21.40	1.06 [0.93, 1.22]	0.88	0.87
GNA	2.54	2.52	*t* = -1.08, *p* = .281	*d*_z_ = -0.05 [-0.14, 0.04]	0.76	0.71	0.64	0.83 [0.71, 0.94]	0.85	0.80

*N* = 1,353 (retrospective before vs. during) and *N* = 446 (T2 vs. during). *M* = mean, *SD* = standard deviation, *M* difference = paired *t*-test, *M* difference *d*_z_ = standardized effect size for the mean differences with bootstrapped 95% CIs. Consistency = Correlation of ratings pertaining to the two timepoints. Variance ratio = variance during COVID-19 restrictions divided by the variance at the respective other timepoint; with bootstrapped 95%-CIs. IC = internal consistency. Internal consistency is calculated as the Spearman-Brown-corrected inter-item correlation for two-item measures (i.e., all but GNA) and as McDonald’s Omega total for multi-item measures (i.e., GNA). SWB = subjective well-being, GNA = general negative appraisal of the COVID-19 pandemic and restrictions. Dut = Duty, Int = Intellect, Adv = Adversity, Mat = Mating, Pos = pOsitivity, Neg = Negativity, Dec = Deception, Soc = Sociality.

#### Well-being

Participants indicated their global life satisfaction (LS) and experienced feelings in February 2020 (i.e., before COVID-19 restrictions, retrospectively assessed), since March 23, 2020 (i.e., during restrictions), and for the four weeks preceding the second assessment (i.e., largely in October 2020 when many restrictions had been lifted). LS was assessed via an 11-point response scale (0 = *completely dissatisfied*; 10 *= completely satisfied*) on a single-item measure [57, 58]. Affect balance (AB) was computed from four items measuring the frequency of experienced feelings (i.e., positive: happy; negative: sad, angry, afraid) with a five-point Likert-type scale (1 = *very rarely*; 5 = *very often*). The mean of the three negative items was then subtracted from the positive item [59]. Measures of subjective well-being (SWB) were obtained by averaging percent-of-maximum-possible transformed LS and AB scores which were highly correlated (before: *r* = .74; during: *r* = .75, follow-up: *r* = .79). The internal consistency of our well-being measure was good (Table 2).

#### General negative appraisal (GNA)

Participants indicated their appraisals of and attitudes towards the pandemic and restrictions by responding to seven items: (a) perceived overall restrictiveness of protective measures, (b) easiness-difficulty of complying with measures, (c) actual compliance with measures, (d) ineffectiveness-effectiveness of measures, (e) perceived leniency-strictness of measures, (f) dissatisfaction-satisfaction with the government response, and (g) pessimism-optimism that the pandemic would be successfully handled. Each item had a different five-point Likert-type response scale. Some items were recoded so that all would be keyed into the same direction (i.e., negative appraisal). We extracted a single component indexing general negative appraisal through principal component analysis (PCA) and used component scores for further statistical analyses. The unweighted sum score of all appraisal items had an acceptable internal consistency (Table 2).

### Statistical analyses

Analyses were carried out using the statistics software R [60]. For the first research question, we examined the mean levels and variances of self-reported situation characteristics for the time before versus during COVID-19 restrictions and for the follow-up assessment versus during COVID-19 restrictions. For the comparison of mean levels, we used paired *t*-tests and report the within-subjects *d*_z_ [61] with bootstrapped 95% confidence intervals (10,000 iterations). For the comparison of variances, a variance ratio was calculated, also with 95% bootstrapped confidence intervals. For the second research question, bivariate Pearson correlations between situation characteristics (retrospective before, during, and follow-up) with personality traits, well-being during COVID-19 restrictions, as well as GNA during COVID-19 restrictions were computed. Prior to the analyses, we specified a set of theoretically plausible personality trait–situation characteristic associations (Table 1) to compare theoretically expected and unexpected effect sizes. In addition to bivariate correlations, we computed multiple regression analyses to examine unique effects of each situation characteristic in the prediction of well-being and GNA during COVID-19 restrictions. All variables were *z*-standardized for multiple regression analyses. Finally, to examine the third research question, we implemented mediation analyses as path models using the R package lavaan [62]. These analyses were used to investigate the degree to which the associations of traits with well-being and GNA during COVID-19 restrictions could be statistically explained by situation characteristics during COVID-19 restrictions. In the first step, individual mediation analyses for the combination of each trait (*n* = 9), situation characteristic (*n* = 8), and dependent variable (*n* = 2) were implemented. In the second step, multiple mediation analyses with all eight situation characteristics as simultaneous mediators were carried out. We focus our interpretation on indirect paths that were (1) statistically significant and (2) had the same direction as a significant total effect. For the multiple mediation analyses, we also examined total indirect effects (through all mediators) and the remaining direct paths from traits to dependent variables. We bootstrapped 95% confidence intervals for all parameter estimates from the mediation analyses using 5,000 iterations. Given the large number of mediation analyses, we focus our interpretations on effects that were significant at the α = .001 level for this specific set of analyses. Again, all variables were *z*-standardized.

A set of robustness analyses was carried out. First, we ensured that our findings were similar when controlling for effects of age and gender. Specifically, we (1) examined the correlations of age and gender with the other variables reported in this study, (2) repeated the multiple regression analyses with age and gender as additional predictors, and (3) repeated the mediation analyses with age and gender as predictors of both the mediators and the outcomes. Second, we examined whether our findings pertaining to mean-level and variance differences in situation experiences and well-being before versus during COVID-19 restrictions were robust to order effects (i.e., whether questions pertaining to before versus during were presented first).

### Open science and transparency

The data from the first assessment have already been used [7], but the current analyses are novel. Our study was not pre-registered a priori and is thus exploratory. However, given our large sample size for most analyses (*N* = 1,353), our effect size estimates are relatively precise, and our statistical power is very high even for small effects (e.g., 95.8% for *r* = .10). Similarly, power for the comparison of situation characteristic mean levels during COVID-19 restrictions versus at follow-up was high even for relatively small effects (*N* = 446, e.g., 88.5% for *d*_z_ = 0.15). The data and all analysis scripts are available on our OSF-page (osf.io/buvp2).

## Results

### Research question 1

**Fig 1:**
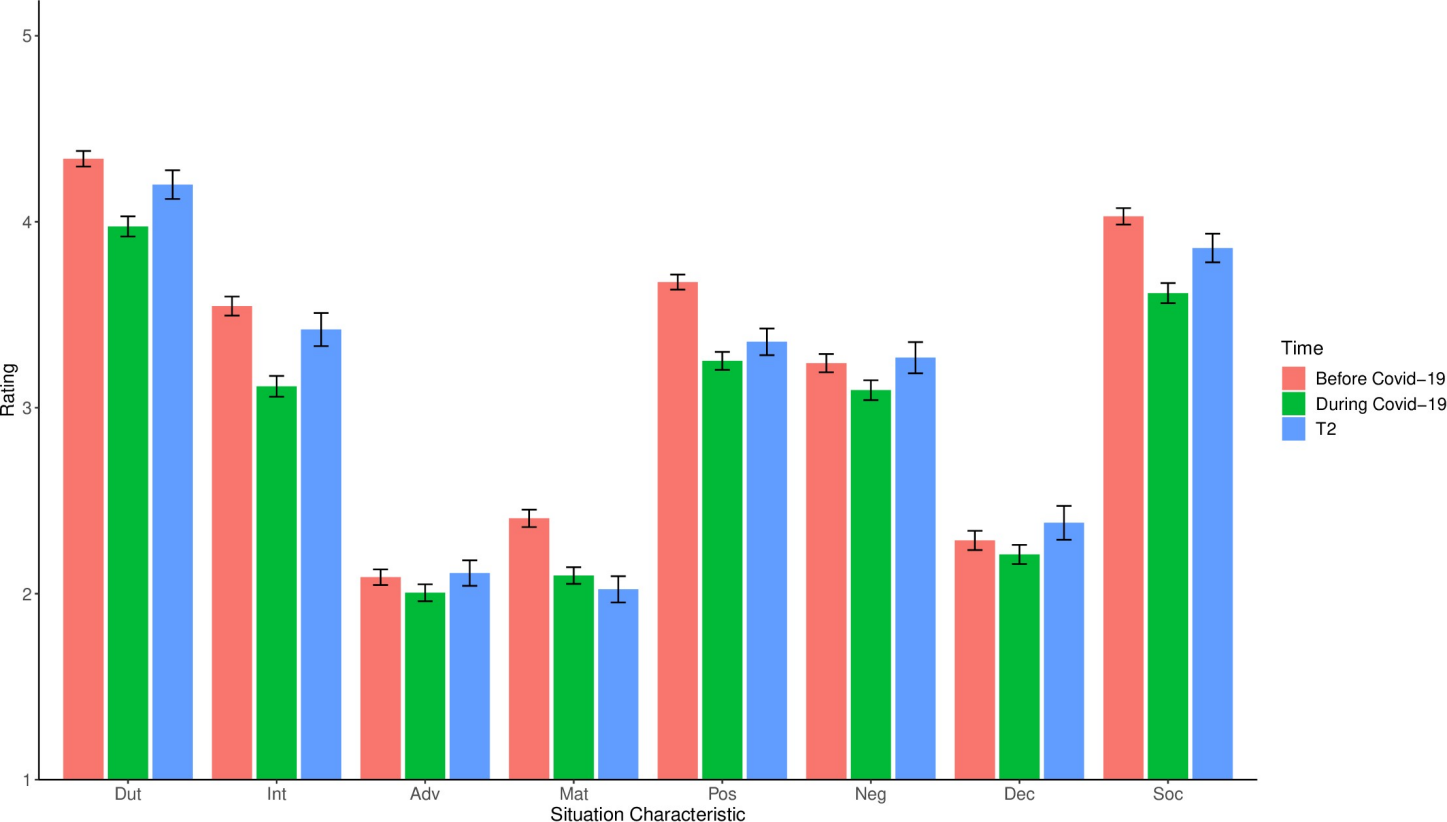
Situation characteristics during COVID-19 restrictions, before (retrospective), and at follow-up. Shown are the means and 95%-CIs for situation characteristics before COVID-19 restrictions (retrospective), during COVID-19 restrictions, and at the follow-up (T2) assessment. *N* = 1,353 (retrospective before, during) and *N* = 446 (T2). Dut = Duty, Int = Intellect, Adv = Adversity, Mat = Mating, Pos = pOsitivity, Neg = Negativity, Dec = Deception, Soc = Sociality.

Fig 1 depicts mean levels and 95%-CIs of self-reported situation characteristics for the three time periods. As can be seen, the profile of situation characteristics was similar across time: People’s everyday situations were on average high in Duty and Sociality; moderate in Intellect, pOsitivity, and Negativity; and low in Adversity, Deception, and Mating. Notwithstanding, some marked mean-level differences between time periods emerged (see Table 2 and Fig 1). Mean levels were significantly lower for all situation characteristics during compared to before COVID-19 restrictions (retrospectively assessed). Similarly, all situation characteristics except Mating were significantly increased at the follow-up assessment compared to during COVID-19 restrictions. The differences before versus during COVID-19 restrictions were most prominent for Duty, Intellect, Mating, pOsitivity, and Sociality (*d*_z_s ≥ 0.41, *p*s < .001) and less pronounced for Adversity, Negativity, and Deception (*d*_z_s ≤ 0.15, *p*s < .001). The differences between situation characteristics at follow-up versus during COVID-19 restrictions were largest for Duty, Intellect, Negativity, and Sociality (*d*_z_s ≥ 0.19, *p*s < .001); less marked for Adversity, pOsitivity, and Deception (*d*_z_s ≤ 0.13, *p*s < .022); and absent for Mating (*d*_z_ = -0.00, *p* = .978). SWB during compared to before COVID-19 restrictions was also markedly lower (*d*_z_ = 0.62, *p* < .001). A similar, albeit less pronounced increase from during COVID-19 restrictions to follow-up was evident (*d*_z_ = 0.20, *p* < .001).

In addition to decreases in mean levels, most situation characteristics showed significant increases in variances from before to during COVID-19 restrictions (see Table 2). The largest increases were found for Duty, pOsitivity, and Sociality with approximately 50% higher between-person variation.

In line with this, all situation characteristics except Deception were also significantly more varied during COVID-19 restrictions versus at follow-up. Again, these differences in variation were largest for Duty, pOsitivity, and Sociality (see Table 2). Similar differences in between-person variation were found for SWB before versus during COVID-19 restrictions, but not at follow-up versus during COVID-19 restrictions. Situation characteristics before (retrospective and during COVID-19 restrictions showed moderate consistency correlations (from *r* = .40 for pOsitivity to *r* = .68 for Deception). This pattern was similar for during ratings versus follow-up (from *r* = .39 for Adversity to *r* = .59 for Intellect). The consistencies of SWB were *r* = .34 (before and during) and *r* = .55 (during and follow-up), respectively.

To gauge the comparability of our measures before COVID-19 restrictions (retrospective) versus at follow-up, we repeated our analyses for these two timepoints. As can be seen in Fig 1, mean levels of situation characteristics before COVID-19 restrictions were generally more similar to the follow-up assessment than to the assessment during COVID-19 restrictions. However, several significant mean-level differences still emerged, largely in the direction of higher situation characteristic scores before COVID-19 restrictions. Most differences in variances before COVID-19 restrictions versus at follow-up were nonsignificant. Correlations between the two timepoints were again substantial (from *r* = .44 for Sociality to *r* = .63 for Intellect). For full results, see S2 Table.

### Research question 2

Table 3 shows the correlations of situation characteristic reports at the three timepoints with personality traits, SWB, and GNA during COVID-19 restrictions. For instance, Extraversion was moderately to strongly related to pOsitivity and Sociality; Openness to Intellect; Neuroticism to Adversity, Negativity, and pOsitivity (reversed); and Machiavellianism to Deception. The average absolute correlations between personality traits and situation characteristics reports were similar for the three time periods: |*r*| = .12, |*r*| = .11, and |*r*| = .12 for before, during, and follow-up, respectively. Importantly, theoretically plausible correlations (see Table 1) were larger on average, with |*r*| = .20, |*r*| = .18, and |*r*| = .18 (expected correlations) versus |*r*| = .08, |*r*| = .08, and |*r*| = .09 (unexpected correlations) for before, during, and follow-up, respectively (see Table 3). Notably, all theoretically plausible correlations had the directions specified in Table 1.

**Table 3:** Correlations with situation experiences.

Timepoint (situation characteristics)	Correlate (during restrictions)	Dut	Int	Adv	Mat	Pos	Neg	Dec	Soc
Before	E	.08 [.03, .14]	.23 [.18, .28]	-.12 [-.17, -.06]	.21 [.16, .26]	.34 [.30, .39]	-.05 [-.10, .00]	-.02 [-.08, .03]	.30 [.25, .35]
	A	.03 [-.03, .08]	.06 [.01, .11]	-.18 [-.23, -.13]	.02 [-.03, .08]	.24 [.19, .29]	-.02 [-.08, .03]	-.20 [-.25, -.14]	.27 [.22, .32]
	C	.14 [.09, .19]	.04 [-.02, .09]	-.22 [-.27, -.16]	.08 [.03, .14]	.17 [.12, .22]	-.10 [-.15, -.05]	-.18 [-.23, -.13]	.10 [.05, .16]
	N	-.02 [-.08, .03]	-.13 [-.18, -.08]	.26 [.21, .31]	-.05 [-.10, .00]	-.37 [-.42, -.33]	.28 [.23, .33]	.04 [-.01, .09]	-.11 [-.16, -.05]
	O	.03 [-.03, .08]	.37 [.33, .42]	-.04 [-.09, .01]	.05 [-.01, .10]	.12 [.07, .17]	.00 [-.05, .06]	.02 [-.04, .07]	.12 [.06, .17]
	H	.04 [-.02, .09]	.07 [.02, .13]	-.06 [-.11, -.01]	-.11 [-.16, -.05]	.07 [.01, .12]	-.04 [-.10, .01]	-.20 [-.25, -.14]	.11 [.06, .16]
	Narc	.03 [-.02, .08]	.22 [.17, .27]	.07 [.02, .13]	.16 [.11, .21]	.02 [-.04, .07]	.16 [.11, .22]	.23 [.18, .28]	.11 [.06, .17]
	Mach	-.02 [-.07, .04]	.06 [.00, .11]	.17 [.11, .22]	.15 [.09, .20]	-.05 [-.10, .00]	.10 [.05, .16]	.36 [.31, .41]	-.04 [-.09, .02]
	Psyc	-.05 [-.10, .01]	-.04 [-.09, .02]	.10 [.05, .15]	.07 [.02, .12]	-.08 [-.14, -.03]	-.03 [-.08, .03]	.22 [.17, .27]	-.17 [-.22, -.12]
	SWB during	.02 [-.04, .07]	.13 [.07, .18]	-.16 [-.21, -.11]	-.06 [-.11, -.01]	.22 [.17, .27]	-.14 [-.19, -.09]	-.01 [-.06, .05]	.00 [-.05, .05]
	GNA_during	.01 [-.04, .07]	-.09 [-.14, -.03]	.10 [.05, .16]	.13 [.08, .18]	.02 [-.04, .07]	-.03 [-.08, .03]	.05 [.00, .10]	.05 [-.01, .10]
During	E	.06 [.01, .12]	.16 [.11, .22]	-.08 [-.14, -.03]	.17 [.12, .22]	.25 [.20, .30]	-.05 [-.10, .00]	.01 [-.05, .06]	.21 [.16, .26]
	A	.00 [-.05, .05]	.07 [.01, .12]	-.15 [-.21, -.10]	.00 [-.06, .05]	.19 [.13, .24]	-.07 [-.12, -.02]	-.17 [-.22, -.11]	.20 [.15, .25]
	C	.14 [.09, .19]	.03 [-.02, .08]	-.17 [-.22, -.12]	.08 [.03, .13]	.16 [.10, .21]	-.09 [-.15, -.04]	-.14 [-.19, -.09]	.10 [.04, .15]
	N	-.05 [-.10, .00]	-.21 [-.26, -.16]	.24 [.19, .29]	-.08 [-.14, -.03]	-.43 [-.47, -.39]	.34 [.29, .38]	.05 [.00, .10]	-.11 [-.16, -.06]
	O	.02 [-.03, .07]	.32 [.27, .37]	-.04 [-.10, .01]	.03 [-.03, .08]	.13 [.07, .18]	-.01 [-.06, .05]	.01 [-.04, .06]	.08 [.03, .13]
	H	.06 [.00, .11]	.06 [.01, .11]	-.08 [-.14, -.03]	-.11 [-.16, -.06]	.09 [.04, .15]	-.05 [-.10, .01]	-.14 [-.19, -.09]	.08 [.03, .13]
	Narc	-.06 [-.11, -.01]	.12 [.07, .17]	.10 [.05, .15]	.10 [.05, .16]	-.04 [-.09, .02]	.11 [.05, .16]	.16 [.11, .22]	.08 [.03, .14]
	Mach	-.02 [-.07, .03]	.06 [.00, .11]	.14 [.08, .19]	.12 [.07, .17]	-.03 [-.09, .02]	.09 [.03, .14]	.30 [.25, .34]	.00 [-.05, .05]
	Psyc	-.04 [-.09, .01]	-.04 [-.09, .02]	.10 [.05, .16]	.10 [.05, .16]	-.12 [-.17, -.07]	.02 [-.03, .07]	.17 [.12, .22]	-.16 [-.21, -.11]
	SWB during	.11 [.06, .16]	.33 [.28, .37]	-.28 [-.33, -.23]	.11 [.06, .16]	.67 [.64, .70]	-.40 [-.45, -.36]	-.05 [-.10, .01]	.20 [.15, .25]
	GNA during	-.06 [-.11, .00]	-.22 [-.27, -.17]	.22 [.17, .27]	.04 [-.02, .09]	-.32 [-.37, -.27]	.20 [.15, .25]	.08 [.02, .13]	-.09 [-.14, -.04]
T2	E	.00 [-.09, .09]	.27 [.18, .35]	-.12 [-.21, -.03]	.15 [.06, .24]	.31 [.22, .39]	-.13 [-.22, -.04]	-.13 [-.22, -.04]	.26 [.17, .35]
	A	-.02 [-.11, .07]	.07 [-.02, .16]	-.19 [-.28, -.10]	.00 [-.09, .10]	.26 [.17, .34]	-.05 [-.15, .04]	-.10 [-.19, -.01]	.24 [.16, .33]
	C	.01 [-.09, .10]	.09 [.00, .18]	-.19 [-.28, -.10]	.10 [.01, .20]	.18 [.08, .26]	-.15 [-.24, -.06]	-.13 [-.22, -.04]	.13 [.03, .22]
	N	.12 [.03, .21]	-.07 [-.16, .02]	.27 [.18, .36]	-.02 [-.11, .07]	-.39 [-.46, -.31]	.37 [.29, .45]	.10 [.01, .19]	-.07 [-.16, .02]
	O	.00 [-.09, .10]	.34 [.25, .42]	-.03 [-.13, .06]	.02 [-.07, .12]	.20 [.11, .29]	-.02 [-.12, .07]	-.01 [-.10, .09]	.20 [.11, .29]
	H	.01 [-.09, .10]	.08 [-.01, .17]	-.09 [-.18, .00]	-.03 [-.13, .06]	.20 [.11, .29]	-.14 [-.23, -.05]	-.22 [-.30, -.12]	.08 [-.02, .17]
	Narc	.00 [-.09, .09]	.18 [.08, .26]	.11 [.02, .20]	.11 [.01, .20]	.01 [-.08, .10]	.10 [.01, .19]	.17 [.08, .26]	.04 [-.06, .13]
	Mach	.02 [-.07, .11]	.03 [-.06, .12]	.14 [.05, .23]	.07 [-.02, .17]	-.09 [-.18, .01]	.04 [-.05, .13]	.29 [.20, .37]	-.07 [-.16, .02]
	Psyc	-.04 [-.13, .05]	-.03 [-.12, .07]	.04 [-.06, .13]	.08 [-.02, .17]	-.12 [-.21, -.02]	.01 [-.08, .10]	.16 [.07, .25]	-.13 [-.22, -.04]
	SWB during	-.06 [-.15, .03]	.12 [.03, .21]	-.13 [-.22, -.04]	-.01 [-.10, .08]	.42 [.34, .50]	-.27 [-.35, -.18]	-.05 [-.14, .05]	.08 [-.02, .17]
	GNA during	.05 [-.04, .14]	-.06 [-.15, .03]	.03 [-.06, .12]	.13 [.04, .22]	-.09 [-.18, .00]	.08 [-.01, .17]	-.01 [-.10, .09]	-.06 [-.15, .03]

*N* = 1,353 (retrospective before and during) and *N* = 446 (T2). Shown are correlations between situation characteristics pertaining to the three timepoints (retrospective before, during, T2) with personality traits, subjective well-being (SWB) during COVID-19 restrictions, and general negative appraisal (GNA) during COVID-19 restrictions. 95% confidence intervals are shown in parentheses. The upper and lower third of the table contain correlations across timepoints to show whether relevant outcomes during COVID-19 restrictions (SWB during, GNA during) correlate more strongly with situation characteristics at the same timepoint than with situation characteristics at the other two timepoints (i.e., indicating time-specific shared variance). Dut = Duty, Int = Intellect, Adv = Adversity, Mat = Mating, Pos = pOsitivity, Neg = Negativity, Dec = Deception, Soc = Sociality, E = Extraversion, A = Agreeableness, C = Conscientiousness, N = Neuroticism, O = Openness, H = Honesty-Humility, Narc = Narcissism, Mach = Machiavellianism, Psyc = Psychopathy.

**Fig 2:**
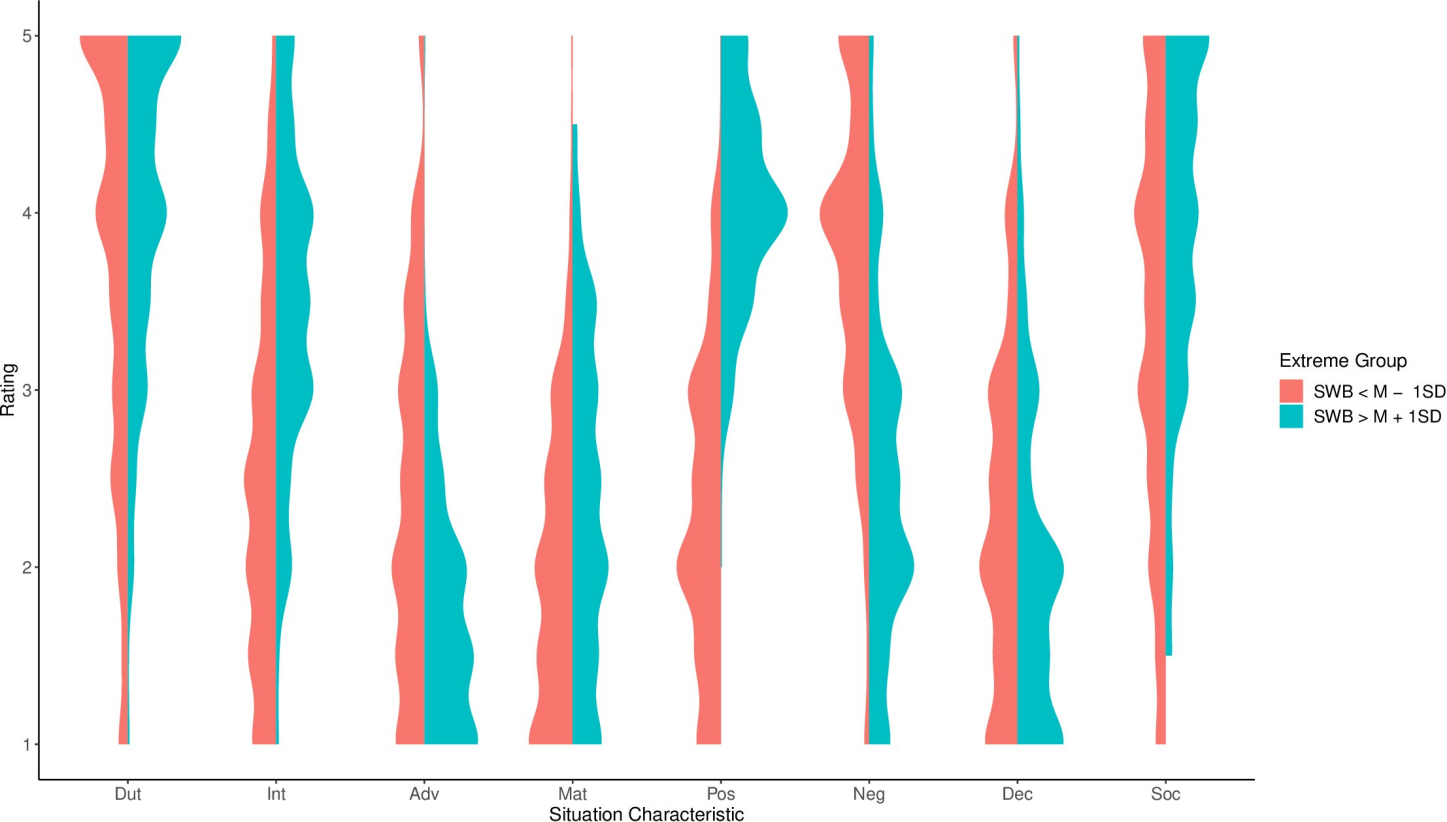
Situation characteristics during the COVID-19 restrictions for extreme groups in well-being. Shown are the smoothed density distributions for situation characteristics during COVID-19 restrictions, separately for participants more than one standard deviation above and below the mean in subjective well-being (SWB), respectively. Dut = Duty, Int = Intellect, Adv = Adversity, Mat = Mating, Pos = pOsitivity, Neg = Negativity, Dec = Deception, Soc = Sociality.

**Fig 3:**
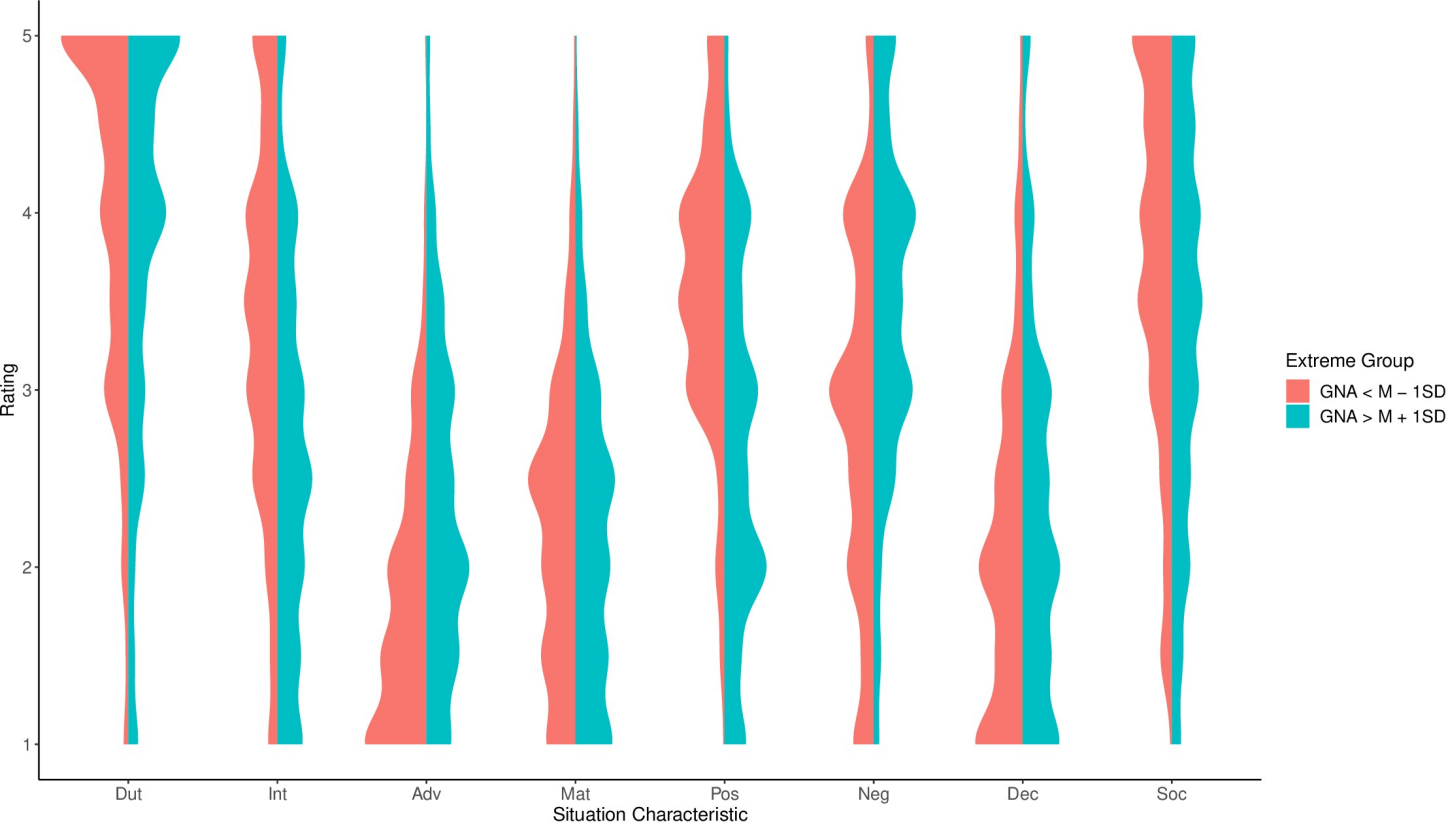
Situation characteristics during the COVID-19 restrictions for extreme groups in general negative appraisal. Shown are the smoothed density distributions for situation characteristics during COVID-19 restrictions, separately for participants more than one standard deviation above and below the mean in general negative appraisal (GNA), respectively. Dut = Duty, Int = Intellect, Adv = Adversity, Mat = Mating, Pos = pOsitivity, Neg = Negativity, Dec = Deception, Soc = Sociality.

Second, SWB and situation characteristics, both pertaining to during COVID-19 restrictions, were meaningfully correlated. Most prominently, higher well-being was associated with more pOsitivity, Intellect, and Sociality and less Negativity and Adversity. Associations between well-being during the COVID-19 restrictions and situation characteristics before the restrictions (retrospective) and at follow-up were similar but much less pronounced, indicating time-specific shared variance (see Table 3). GNA during COVID-19 restrictions was also meaningfully associated with situation characteristics during COVID-19 restrictions. In particular, more negative appraisal was associated with less pOsitivity and Intellect, and more Adversity and Negativity. Associations of GNA with situation characteristics before COVID-19 restrictions and at follow-up were much smaller, again indicating time-specific shared variance (Table 3). Associations between situation characteristic reports during the COVID-19 restrictions with well-being as well as GNA during COVID-19 restrictions are visualized using situation profiles of extreme groups in the outcome variables (see Figs 2 and 3).

Finally, we ran multiple regression analyses with all eight situation characteristics during the restrictions in the prediction of SWB and GNA during COVID-19 restrictions (Table 4). After controlling for all other situation characteristics, only Duty, Intellect, Adversity, pOsitivity, and Negativity significantly predicted well-being, with the largest effect size for pOsitivity. Moreover, Intellect, Adversity, Mating, pOsitivity, and Negativity had significant unique effects in the prediction of GNA, with the largest (negative) effect size again for pOsitivity.

**Table 4:** Multiple regression analyses.

DV	Predictor	β	95%-CI	*t*	*p*
SWB during					
	Dut	.05	[.00, .09]	2.11	.035
	Int	.13	[.08, .17]	5.83	< .001
	Adv	-.09	[-.13, -.05]	-4.22	< .001
	Mat	-.02	[-.05, .02]	-0.77	.439
	Pos	.53	[.49, .58]	21.91	< .001
	Neg	-.22	[-.27, -.18]	-9.48	< .001
	Dec	.02	[-.01, .06]	1.22	.222
	Soc	-.02	[-.07, .02]	-1.05	.293
GNA during					
	Dut	-.01	[-.07, .04]	-0.52	.603
	Int	-.16	[-.21, -.10]	-5.43	< .001
	Adv	.13	[.07, .18]	4.61	< .001
	Mat	.10	[.04, .15]	3.64	< .001
	Pos	-.23	[-.29, -.17]	-7.24	< .001
	Neg	.08	[.02, .14]	2.55	.011
	Dec	.04	[-.01, .09]	1.55	.121
	Soc	.02	[-.04, .07]	0.59	.558

*N* = 1,353. Multiple regression analyses with situation characteristics during COVID-19 restrictions predicting subjective well-being (SWB) and general negative appraisal (GNA) during COVID-19 restrictions. Dut = Duty, Int = Intellect, Adv = Adversity, Mat = Mating, Pos = pOsitivity, Neg = Negativity, Dec = Deception, Soc = Sociality.

### Research question 3

Mediation analyses with one trait as independent variable and one situation characteristic as mediator were carried out (see Table 5). In the first step, we examined the total effects of personality traits on well-being. They were significant at α = .001 for Extraversion, Agreeableness, Conscientiousness, and Neuroticism (|*r*s| > .10, *p*s < .001). In the second step, we examined significant indirect effects that had the same direction as a significant total effect (bold in Table 5). Intellect, Adversity, pOsitivity, Negativity, and Sociality statistically mediated the effect of at least one trait at α = .001, with the largest effect sizes for pOsitivity. Regarding GNA, significant total effects were observed for Agreeableness, Neuroticism, Honesty-Humility, and Psychopathy (|*r*s| > .10, *p*s < .001). Intellect, Adversity, pOsitivity, and Negativity statistically mediated the effect of at least one trait at α = .001.

**Table 5:** Mediation analyses.

	Trait	Total	Indirect effect via							
			Dut	Int	Adv	Mat	Pos	Neg	Dec	Soc
SWB during										
	E	**.19 [.13, .24]**	.01 [.00, .01]	**.05 [.03, .07]**	.02 [.01, .04]	.01 [.00, .03]	**.16 [.13, .20]**	.02 [.00, .04]	.00 [.00, .00]	**.04 [.02, .05]**
	A	**.13 [.07, .18]**	.00 [-.01, .01]	.02 [.00, .04]	**.04 [.02, .06]**	.00 [-.01, .01]	**.12 [.09, .16]**	.03 [.01, .05]	.00 [-.01, .01]	**.04 [.02, .05]**
	C	**.13 [.07, .18]**	.01 [.00, .02]	.01 [-.01, .03]	**.05 [.03, .06]**	.01 [.00, .02]	**.10 [.07, .14]**	.04 [.01, .06]	.00 [.00, .01]	.02 [.01, .03]
	N	**-.50 [-.54, -.45]**	.00 [-.01, .00]	**-.05 [-.07, -.03]**	**-.04 [-.06, -.03]**	-.01 [-.01, .00]	**-.24 [-.27, -.21]**	**-.09 [-.11, -.07]**	.00 [.00, .00]	-.02 [-.03, -.01]
	O	.10 [.04, .16]	.00 [.00, .01]	.11 [.08, .13]	.01 [.00, .03]	.00 [.00, .01]	.08 [.05, .12]	.00 [-.02, .02]	.00 [.00, .00]	.02 [.00, .03]
	H	.08 [.02, .13]	.01 [.00, .01]	.02 [.00, .04]	.02 [.01, .04]	-.01 [-.02, -.01]	.06 [.02, .10]	.02 [.00, .04]	.00 [.00, .01]	.02 [.00, .03]
	Narc	-.04 [-.09, .02]	-.01 [-.01, .00]	.04 [.02, .06]	-.03 [-.04, -.01]	.01 [.00, .02]	-.02 [-.06, .01]	-.04 [-.07, -.02]	-.01 [-.02, .00]	.02 [.01, .03]
	Mach	-.04 [-.09, .01]	.00 [-.01, .00]	.02 [.00, .04]	-.04 [-.06, -.02]	.01 [.01, .02]	-.02 [-.06, .01]	-.04 [-.06, -.01]	-.01 [-.03, .01]	.00 [-.01, .01]
	Psyc	-.07 [-.12, -.01]	.00 [-.01, .00]	-.01 [-.03, .01]	-.03 [-.05, -.01]	.01 [.00, .02]	-.08 [-.12, -.04]	-.01 [-.03, .01]	-.01 [-.02, .00]	-.03 [-.05, -.02]
GNA during										
	E	.02 [-.04, .08]	.00 [-.01, .00]	-.04 [-.06, -.02]	-.02 [-.03, -.01]	.01 [.00, .02]	-.09 [-.11, -.06]	-.01 [-.02, .00]	.00 [.00, .01]	-.02 [-.04, -.01]
	A	**-.16 [-.22, -.10]**	.00 [.00, .00]	-.01 [-.03, .00]	**-.03 [-.05, -.02]**	.00 [.00, .00]	**-.06 [-.08, -.04]**	-.01 [-.02, .00]	-.01 [-.02, .00]	-.01 [-.03, .00]
	C	-.06 [-.11, .00]	-.01 [-.02, .00]	-.01 [-.02, .01]	-.04 [-.06, -.02]	.00 [.00, .01]	-.05 [-.07, -.03]	-.02 [-.03, -.01]	-.01 [-.02, .00]	-.01 [-.02, .00]
	N	**.12 [.06, .18]**	.00 [.00, .01]	**.04 [.03, .06]**	**.05 [.03, .07]**	.00 [-.01, .00]	**.14 [.11, .18]**	**.06 [.04, .08]**	.00 [.00, .01]	.01 [.00, .02]
	O	-.09 [-.14, -.03]	.00 [-.01, .00]	-.07 [-.09, -.05]	-.01 [-.02, .00]	.00 [.00, .00]	-.04 [-.06, -.02]	.00 [-.01, .01]	.00 [.00, .01]	-.01 [-.01, .00]
	H	**-.12 [-.18, -.05]**	.00 [-.01, .00]	-.01 [-.03, .00]	-.02 [-.03, -.01]	.00 [-.01, .00]	-.03 [-.05, -.01]	-.01 [-.02, .00]	-.01 [-.02, .00]	-.01 [-.01, .00]
	Narc	.01 [-.05, .07]	.00 [.00, .01]	-.03 [-.04, -.01]	.02 [.01, .04]	.00 [.00, .01]	.01 [-.01, .03]	.02 [.01, .03]	.01 [.00, .03]	-.01 [-.02, .00]
	Mach	.06 [.00, .12]	.00 [.00, .01]	-.01 [-.03, .00]	.03 [.02, .04]	.00 [.00, .01]	.01 [-.01, .03]	.02 [.01, .03]	.02 [.00, .04]	.00 [-.01, .01]
	Psyc	**.19 [.13, .25]**	.00 [.00, .01]	.01 [.00, .02]	.02 [.01, .04]	.00 [.00, .01]	**.04 [.02, .05]**	.00 [-.01, .02]	.01 [.00, .02]	.01 [.00, .02]

*N* = 1,353. Presented are the simple mediation analyses. One mediation model for each trait–situation characteristic–dependent variable combination was fitted. Total = direct effect in a model without a mediator. Indirect effect via = indirect effect mediated via individual situation characteristics. 95%-CIs are given in parentheses. Direct effects printed in bold are significant at α = .001. Indirect effects are printed in bold if they are significant at α = .001, have the same direction as the total effect, and the total effect is significant at α = .001. Direct effects when controlling for each individual mediator were omitted but can be found at osf.io/buvp2. SWB = subjective well-being, GNA = general negative appraisal. Dut = Duty, Int = Intellect, Adv = Adversity, Mat = Mating, Pos = pOsitivity, Neg = Negativity, Dec = Deception, Soc = Sociality, E = Extraversion, A = Agreeableness, C = Conscientiousness, N = Neuroticism, O = Openness, H = Honesty-Humility, Narc = Narcissism, Mach = Machiavellianism, Psyc = Psychopathy.

These analyses were followed up by multiple mediation analyses in which all eight situation characteristics served as simultaneous mediators (see Table 6). For these analyses, we examined (1) the total indirect effects and the remaining direct effects, and (2) significant unique indirect effects of individual situation characteristics with the same direction as a significant total effect. Regarding well-being, substantial total indirect effects were observed for all traits with significant total effects (*p*s < .001). The remaining direct effects of all traits except Neuroticism on well-being were reduced to essentially zero and were no longer significant. The direct effect of Neuroticism was more than halved after statistically accounting for situation characteristics reports. Intellect, pOsitivity, and Negativity emerged as mediators for at least one trait, with the largest indirect effects for pOsitivity. With respect to GNA, pronounced total indirect effects were again observed. The following patterns of findings emerged: The direct effect was (a) reduced but still significant (Psychopathy), (b) reduced and no longer significant (at α = .001; Agreeableness, Honesty-Humility), (c) descriptively even reversed (Neuroticism), or (d) not significant in the model without mediators (Extraversion, Conscientiousness, Openness, Narcissism, Machiavellianism). Significant indirect effects were observed for Intellect, Adversity, and pOsitivity, again with the largest effects for pOsitivity (see Table 6). More details on the results of our mediation analyses can be found at osf.io/buvp2.

**Table 6:** Multiple mediation analyses.

	Trait	Total	Direct	Indirect total	Indirect effect via							
					Dut	Int	Adv	Mat	Pos	Neg	Dec	Soc
SWB during	E	**.19 [.13, .24]**	.02 [-.02, .06]	**.17 [.13, .21]**	.00 [.00, .01]	**.02 [.01, .03]**	.01 [.00, .01]	.00 [-.01, .00]	**.13 [.10, .16]**	.01 [.00, .02]	.00 [.00, .00]	-.01 [-.02, .00]
	A	**.13 [.07, .18]**	.00 [-.04, .04]	**.13 [.09, .17]**	.00 [.00, .00]	.01 [.00, .02]	.01 [.01, .02]	.00 [.00, .00]	**.10 [.07, .13]**	.02 [.00, .03]	.00 [-.01, .00]	.00 [-.01, .00]
	C	**.13 [.07, .18]**	.01 [-.03, .05]	**.12 [.08, .16]**	.01 [.00, .01]	.00 [.00, .01]	.01 [.01, .02]	.00 [-.01, .00]	**.08 [.05, .11]**	.02 [.01, .03]	.00 [-.01, .00]	.00 [-.01, .00]
	N	**-.50 [-.54, -.45]**	**-.19 [-.24, -.15]**	**-.30 [-.34, -.27]**	.00 [-.01, .00]	**-.02 [-.03, -.01]**	-.02 [-.03, -.01]	.00 [.00, .01]	**-.20 [-.24, -.17]**	**-.06 [-.08, -.04]**	.00 [.00, .00]	.00 [.00, .01]
	O	.10 [.04, .16]	-.02 [-.06, .02]	.12 [.07, .16]	.00 [.00, .00]	.04 [.03, .06]	.00 [.00, .01]	.00 [.00, .00]	.07 [.04, .10]	.00 [-.01, .01]	.00 [.00, .00]	.00 [-.01, .00]
	H	.08 [.02, .13]	.00 [-.04, .04]	.07 [.03, .12]	.00 [.00, .01]	.01 [.00, .02]	.01 [.00, .01]	.00 [.00, .01]	.05 [.02, .08]	.01 [.00, .02]	.00 [-.01, .00]	.00 [-.01, .00]
	Narc	-.04 [-.09, .02]	.00 [-.04, .04]	-.04 [-.08, .00]	.00 [-.01, .00]	.02 [.01, .02]	-.01 [-.02, .00]	.00 [-.01, .00]	-.02 [-.05, .01]	-.02 [-.04, -.01]	.00 [.00, .01]	.00 [-.01, .00]
	Mach	-.04 [-.09, .01]	.00 [-.04, .04]	-.04 [-.08, .00]	.00 [.00, .00]	.01 [.00, .01]	-.01 [-.02, -.01]	.00 [-.01, .00]	-.02 [-.05, .01]	-.02 [-.03, -.01]	.01 [.00, .02]	.00 [.00, .00]
	Psyc	-.07 [-.12, -.01]	.01 [-.03, .05]	-.08 [-.12, -.04]	.00 [-.01, .00]	.00 [-.01, .00]	-.01 [-.02, .00]	.00 [-.01, .00]	-.06 [-.09, -.03]	.00 [-.02, .01]	.00 [.00, .01]	.00 [.00, .01]
GNA during	E	.02 [-.04, .08]	**.11 [.06, .16]**	-.09 [-.12, -.06]	.00 [-.01, .00]	-.03 [-.04, -.01]	-.01 [-.02, .00]	.01 [.00, .03]	-.06 [-.08, -.04]	.00 [-.01, .00]	.00 [.00, .00]	.00 [-.01, .01]
	A	**-.16 [-.22, -.10]**	-.09 [-.15, -.04]	**-.07 [-.10, -.05]**	.00 [.00, .00]	-.01 [-.02, .00]	-.02 [-.03, -.01]	.00 [-.01, .00]	**-.04 [-.06, -.03]**	-.01 [-.01, .00]	.00 [-.01, .00]	.01 [-.01, .02]
	C	-.06 [-.11, .00]	.01 [-.04, .07]	-.07 [-.10, -.04]	.00 [-.01, .01]	.00 [-.01, .00]	-.02 [-.04, -.01]	.01 [.00, .02]	-.04 [-.05, -.02]	-.01 [-.02, .00]	-.01 [-.01, .00]	.00 [.00, .01]
	N	**.12 [.06, .18]**	-.08 [-.14, -.03]	**.20 [.16, .24]**	.00 [.00, .01]	**.03 [.02, .05]**	**.03 [.02, .05]**	-.01 [-.02, .00]	**.11 [.08, .14]**	.03 [.01, .06]	.00 [.00, .01]	.00 [-.01, .00]
	O	-.09 [-.14, -.03]	.00 [-.06, .05]	-.08 [-.11, -.05]	.00 [.00, .00]	-.05 [-.07, -.03]	-.01 [-.01, .00]	.00 [.00, .01]	-.03 [-.05, -.02]	.00 [-.01, .00]	.00 [.00, .00]	.00 [.00, .01]
	H	**-.12 [-.18, -.05]**	-.06 [-.11, .00]	**-.06 [-.08, -.03]**	.00 [-.01, .00]	-.01 [-.02, .00]	-.01 [-.02, .00]	-.01 [-.02, .00]	-.02 [-.04, -.01]	.00 [-.01, .00]	.00 [-.01, .00]	.00 [.00, .01]
	Narc	.01 [-.05, .07]	-.02 [-.08, .03]	.03 [.00, .06]	.00 [.00, .01]	-.02 [-.03, -.01]	.01 [.01, .02]	.01 [.00, .02]	.01 [-.01, .02]	.01 [.00, .02]	.01 [.00, .02]	.00 [.00, .01]
	Mach	.06 [.00, .12]	.01 [-.04, .07]	.05 [.02, .08]	.00 [.00, .00]	-.01 [-.02, .00]	.02 [.01, .03]	.01 [.00, .02]	.01 [-.01, .02]	.01 [.00, .02]	.01 [-.01, .03]	.00 [.00, .00]
	Psyc	**.19 [.13, .25]**	**.14 [.09, .20]**	**.05 [.02, .08]**	.00 [.00, .00]	.01 [.00, .02]	.01 [.00, .02]	.01 [.00, .02]	**.03 [.01, .04]**	.00 [.00, .01]	.00 [-.01, .01]	-.01 [-.02, .00]

*N* = 1,353. Presented are multiple mediation analyses. One mediation model for each trait–dependent variable combination was fitted. Total = direct effect in a model without mediators. Direct = direct effect remaining in models with all eight mediators included. Indirect effect via = indirect effect mediated via situation characteristics. 95%-CIs are given in parentheses. Direct effects printed in bold are significant at α = .001. Indirect effects are printed in bold if they are significant at α = .001, have the same direction as the total effect, and the total effect is significant at α = .001. More detailed models can be found at osf.io/buvp2. SWB = subjective well-being, GNA = general negative appraisal. Dut = Duty, Int = Intellect, Adv = Adversity, Mat = Mating, Pos = pOsitivity, Neg = Negativity, Dec = Deception, Soc = Sociality, E = Extraversion, A = Agreeableness, C = Conscientiousness, N = Neuroticism, O = Openness, H = Honesty-Humility, Narc = Narcissism, Mach = Machiavellianism, Psyc = Psychopathy.

### Robustness analyses

S3 Table depicts correlations with age and gender. In the multiple regression analyses with age and gender as additional predictors, the pattern of effects remained identical and the regression coefficients changed only slightly (S4 Table). The pattern of results from the mediation analyses also remained largely identical (see S5 and S6 Tables and for more details osf.io/buvp2). Thus, our results are robust when controlling for the demographic variables age and gender.

Furthermore, our results pertaining to the mean and variance differences in situation characteristics and well-being before versus during COVID-19 restrictions were similar for the two orders of presentation (before first vs. during first). While small order effects emerged for during-related ratings, most importantly, the results were similar to the main analyses when examining only the questions presented first (i.e., avoiding possible contrast effects; for full details see S7 Table).

## Discussion

### Summary and interpretation

The present study sought to further our understanding of person-environment relations during the COVID-19 crisis in Germany. To this end, we focused on participants’ situation experiences. First, we found that mean levels of most situation characteristic reports were lower during compared to before (retrospective) the implementation of COVID-19 restrictions and during the restrictions compared to follow-up. Across the two comparisons, means were especially lower during COVID-19 restrictions for Duty, Intellect, and Sociality which is expected given the implemented restrictions (e.g., several businesses and offices as well as universities and schools were closed, social distancing was implemented). SWB was also lower during COVID-19 restrictions compared to before (retrospective) and at follow-up. These findings are partly corroborated by longitudinal work on mental health and well-being change during COVID-19 (e.g., [63, 64], cf. [65], see also [4, 66]).

Second, between-person variances in most situation characteristics reports were elevated during COVID-19 restrictions compared to before and follow-up, especially for Duty, pOsitivity, and Sociality (approximately 50% more variation). This may be attributable to heterogeneous situation change trajectories due to restrictions (e.g., some people continue going to work while others can no longer work or work from home; some people become socially isolated while others are permanently with their family members during the crisis) and individual differences in construal and coping during the crisis.

Third, situation experiences were correlated with personality traits in theoretically expected ways, in line with our hypotheses (e.g., [25]), and corroborating and extending previous work (e.g., by also investigating Dark Triad traits and well-being). This was the case for situation characteristics pertaining to all three timepoints. Hence, COVID-19 –representing a very pronounced change to persons’ environments–did not eradicate person-environment correlations [10]. These correlations could be attributable to both situation contact (i.e., different trait levels encounter different objective situations) and situation construal (i.e., trait levels affect idiosyncratic situation perception; see [17]). Further, situation experiences were meaningfully associated with both well-being during and appraisal of the COVID-19 situation. Psycho-social impacts of the COVID-19 crisis were thus varied, and long-term effects may transform people’s socio-ecological niches and their mental health with both adverse and beneficial consequences [67].

Lastly, personality correlated in meaningful ways with both well-being and negative appraisal (see [7]). Interestingly, the relationship between personality traits and well-being during COVID-19 restrictions was statistically fully mediated by situation experiences for all traits except Neuroticism (where still more than half of the total effect was mediated). This pattern was slightly less pronounced for GNA, but substantial total indirect effects emerged here as well. Across both single and multiple mediation analyses, Intellect, pOsitivity, and Negativity (well-being) as well as Intellect, Adversity, and pOsitivity (GNA) emerged as statistical mediators. Thus, the valence of situation experiences appears to be crucial in both cases. As Horstmann and colleagues [16] have shown, such valence effects may not be fully reduced to affect confounds but are likely substantive. Moreover, some patterns above and beyond valence also emerged in the multiple mediation analyses (e.g., indirect effects via Intellect).

### Limitations and outlook

First, we only used self-report variables in this study, and it may be desirable to extend this work by incorporating other-reports. Second, we could not distinguish between situation contact (i.e., actual situations) and situation construal (i.e., unique perceptions). Although previous work indicates that person-situation relations may largely reside at the construal level [17, 35], this need not be the case during the COVID-19 crisis where several objective changes to people’s lives (that likely differ between individuals) occurred. Third, our sample is not representative for Germany, although it is substantially more diverse than frequently used undergraduate samples. Fourth, the retrospective nature of our measures pertaining to the time period before COVID-19 is an important limitation and mean differences may reflect subjectively perceived rather than actual change [27]. Nevertheless, the observed effects were to a large degree corroborated by our follow-up assessment. Here, it should be noted as a limitation that temporal effects other than the change in COVID-19 restrictions (e.g., seasonality) may have partly affected observed changes which can therefore only tentatively be attributed to loosened restrictions. Moreover, some mean-level differences between the retrospective assessment before COVID-19 restrictions and the follow-up assessment were found, although it is unclear whether these are attributable to methodological factors or actual differences between the two timepoints (e.g., it could be argued that the psychological situations at follow-up did not fully return to baseline given the ongoing pandemic despite loosened restrictions at this time). Finally, no causal interpretations with respect to the mediation analyses are permitted given the observational and cross-sectional nature of the data used for these analyses. It would have been preferable to manipulate the mediating mechanisms (i.e., the situation experiences) to examine effects on well-being. However, this was not possible with the backdrop of the COVID-19 pandemic. Future work may extend our findings, for instance using experimental manipulations of participants’ situations.

## Conclusion

Overall, our findings indicate that situation experiences are important individual difference variables that are meaningfully associated with outcome variables. Moreover, they are substantially associated with traits and may partly explain the links between traits and outcome variables. Examining person-environment relations during the COVID-19 crisis yields a more complete picture of human functioning during the pandemic.

## Supporting information

S1 Table Descriptives of the trait measures. (DOCX)

S2 Table Descriptive statistics of situation characteristics (retrospective pre vs. follow-up). (DOCX)

S3 Table Correlations with age and gender. (DOCX)

S4 Table Multiple regression analyses with age and gender as additional predictors. (DOCX)

S5 Table Mediation analyses with age and gender as additional predictors of the mediator and outcome. (DOCX)

S6 Table Multiple mediation analyses with age and gender as additional predictors of each mediator and the outcome. (DOCX)

S7 Table Robustness analyses with respect to order effects. (DOCX)
