# Supplementary material for: The situation during the COVID-19 pandemic: A snapshot in Germany
Source: PLoS One. 2021 Feb 12;16(2):e0245719. doi: 10.1371/journal.pone.0245719 (PMC7880467; doi:10.1371/journal.pone.0245719)
Supplement: S1 Table — (DOCX) [file pone.0245719.s001.docx]

**S1 Table. Descriptives of the trait measures**

|  | *M* | *SD* | ω | α |
| --- | --- | --- | --- | --- |
| E | 3.20 | 0.72 | .73 | .73 |
| A | 3.74 | 0.59 | .69 | .68 |
| C | 3.58 | 0.70 | .76 | .75 |
| N | 2.76 | 0.81 | .82 | .82 |
| O | 3.69 | 0.70 | .72 | .72 |
| H | 3.63 | 0.65 | .54 | .53 |
| Narc | 4.25 | 2.03 | .84 | .84 |
| Mach | 2.88 | 1.70 | .76 | .76 |
| Psyc | 2.70 | 1.53 | .59 | .59 |

*N* = 1,353. Shown are the internal consistencies and descriptives of the trait measures used in this study. α = Cronbach’s Alpha. ω = McDonald’s Omega total. E = Extraversion, A = Agreeableness, C = Conscientiousness, N = Neuroticism, O = Openness, H = Honesty-Humility, Narc = Narcissism, Mach = Machiavellianism, Psyc = Psychopathy.
