# Supplementary material for: The situation during the COVID-19 pandemic: A snapshot in Germany
Source: PLoS One. 2021 Feb 12;16(2):e0245719. doi: 10.1371/journal.pone.0245719 (PMC7880467; doi:10.1371/journal.pone.0245719)
Supplement: S2 Table — (DOCX) [file pone.0245719.s002.docx]

**S2 Table. Descriptive Statistics of Situation Characteristics (retrospective pre vs. follow-up)**

|  | *M*  T2 | *M*  before | *M*  difference | *M*  difference *d*_z_ | Consistency  *r* | *SD*  T2 | *SD*  before | Variance  Ratio | IC  T2 | IC  before |
| --- | --- | --- | --- | --- | --- | --- | --- | --- | --- | --- |
| dut | 4.20 | 4.37 | *t* = 4.77, *p* < .001 | *d*_z_ = 0.23[0.13,0.32] | 0.53 | 0.83 | 0.76 | 0.83[0.68,1.02] | 0.80 | 0.86 |
| int | 3.42 | 3.67 | *t* = 6.38, *p* < .001 | *d*_z_ = 0.30[0.21,0.40] | 0.63 | 0.96 | 0.95 | 0.99[0.86,1.13] | 0.78 | 0.84 |
| adv | 2.11 | 2.07 | *t* = -1.14, *p* = .253 | *d*_z_ = -0.05[-0.15,0.04] | 0.48 | 0.74 | 0.76 | 1.06[0.88,1.28] | 0.65 | 0.70 |
| mat | 2.02 | 2.32 | *t* = 7.33, *p* < .001 | *d*_z_ = 0.35[0.26,0.44] | 0.46 | 0.76 | 0.87 | 1.31[1.13,1.53] | 0.40 | 0.40 |
| pos | 3.35 | 3.69 | *t* = 9.56, *p* < .001 | *d*_z_ = 0.45[0.36,0.55] | 0.54 | 0.77 | 0.77 | 1.01[0.86,1.19] | 0.73 | 0.79 |
| neg | 3.27 | 3.24 | *t* = -0.61, *p* = .541 | *d*_z_ = -0.03[-0.12,0.06] | 0.45 | 0.90 | 0.94 | 1.08[0.93,1.25] | 0.74 | 0.79 |
| dec | 2.38 | 2.35 | *t* = -0.79, *p* = .430 | *d*_z_ = -0.04[-0.13,0.06] | 0.51 | 0.98 | 0.97 | 0.98[0.84,1.14] | 0.78 | 0.80 |
| soc | 3.86 | 4.05 | *t* = 4.63, *p* < .001 | *d*_z_ = 0.22[0.13,0.31] | 0.44 | 0.83 | 0.81 | 0.95[0.80,1.13] | 0.51 | 0.65 |
| SWB | 62.01 | 71.62 | *t* = 10.15, *p* < .001 | *d*_z_ = 0.48[0.38,0.58] | 0.45 | 20.78 | 16.87 | 0.66[0.55,0.78] | 0.88 | 0.85 |

*N* = 446. *M* = mean, *SD* = standard deviation, *M* difference = paired *t*-test, *M* difference *d*_z_ = standardized effect size for the mean differences with bootstrapped 95% CIs. Consistency = Correlation of ratings pertaining to the two timepoints. Variance ratio = variance before COVID-19 restrictions (retrospective) divided by the variance at the follow-up assessment; with bootstrapped 95%-CIs. IC = internal consistency. Internal consistency is calculated as the Spearman-Brown-corrected inter-item correlation. SWB = subjective well-being. Dut = Duty, Int = Intellect, Adv = Adversity, Mat = Mating, Pos = pOsitivity, Neg = Negativity, Dec = Deception, Soc = Sociality.
