# Supplementary material for: The situation during the COVID-19 pandemic: A snapshot in Germany
Source: PLoS One. 2021 Feb 12;16(2):e0245719. doi: 10.1371/journal.pone.0245719 (PMC7880467; doi:10.1371/journal.pone.0245719)
Supplement: S3 Table — (DOCX) [file pone.0245719.s003.docx]

**S3 Table. Correlations with age and gender**

|  | age | gender |
| --- | --- | --- |
| Dut | -.01 [-.06, .05] | -.14 [-.19, -.08] |
| Int | -.05 [-.11, .00] | .01 [-.04, .06] |
| Adv | -.06 [-.11, .00] | .07 [.02, .12] |
| Mat | -.10 [-.15, -.05] | .07 [.01, .12] |
| Pos | -.01 [-.06, .04] | -.11 [-.16, -.06] |
| Neg | -.14 [-.20, -.09] | -.08 [-.14, -.03] |
| Dec | -.10 [-.15, -.04] | .15 [.10, .21] |
| Soc | -.06 [-.12, -.01] | -.14 [-.19, -.08] |
| E | -.02 [-.08, .03] | -.02 [-.07, .04] |
| A | -.04 [-.09, .01] | -.21 [-.26, -.16] |
| C | .07 [.02, .13] | -.14 [-.20, -.09] |
| N | -.10 [-.16, -.05] | -.10 [-.16, -.05] |
| O | -.02 [-.08, .03] | .05 [-.01, .10] |
| H | .10 [.05, .15] | -.16 [-.21, -.11] |
| Narc | -.23 [-.28, -.18] | .06 [.01, .11] |
| Mach | -.13 [-.18, -.08] | .13 [.08, .18] |
| Psyc | -.05 [-.11, .00] | .27 [.22, .32] |
| SWB during | .01 [-.04, .06] | -.01 [-.06, .04] |
| GNA during | -.04 [-.10, .01] | .11 [.06, .16] |

*N* = 1,353. Shown are the correlations between situation characteristics

during COVID-19 restrictions, personality, subjective well-being (SWB) during

COVID-19 restrictions, and general negative appraisal (GNA) during COVID-19
restrictions with age and gender (1 = male). 95% confidence intervals are shown
in parentheses. Dut = Duty, Int =Intellect, Adv = Adversity, Mat = Mating,
Pos = pOsitivity, Neg = Negativity, Dec = Deception, Soc = Sociality,
E = Extraversion, A = Agreeableness, C = Conscientiousness, N = Neuroticism,
O = Openness, H = Honesty-Humility, Narc = Narcissism, Mach = Machiavellianism,

Psyc = Psychopathy.
