# Supplementary material for: The situation during the COVID-19 pandemic: A snapshot in Germany
Source: PLoS One. 2021 Feb 12;16(2):e0245719. doi: 10.1371/journal.pone.0245719 (PMC7880467; doi:10.1371/journal.pone.0245719)
Supplement: S4 Table — (DOCX) [file pone.0245719.s004.docx]

**S4 Table. Multiple regression analyses with age and gender as additional predictors.**

| DV | Predictor | β | 95%-CI | *t* | *p* |
| --- | --- | --- | --- | --- | --- |
| SWB during |  |  |  |  |  |
|  | Dut | .05 | [ .01, .09] | 2.33 | .020 |
|  | Int | .12 | [ .08, .17] | 5.61 | <.001 |
|  | Adv | -.09 | [-.13, -.05] | -4.36 | <.001 |
|  | Mat | -.02 | [-.06, .02] | -1.05 | .296 |
|  | Pos | .54 | [ .49, .59] | 21.99 | <.001 |
|  | Neg | -.22 | [-.26, -.17] | -9.21 | <.001 |
|  | Dec | .02 | [-.02, .06] | 0.82 | .413 |
|  | Soc | -.02 | [-.06, .02] | -0.85 | .394 |
|  | age | -.02 | [-.06, .02] | -1.05 | .295 |
|  | gender | .08 | [ .00, .17] | 2.06 | .039 |
| GNA during |  |  |  |  |  |
|  | Dut | -.01 | [-.06, .05] | -0.19 | .853 |
|  | Int | -.16 | [-.22, -.11] | -5.73 | <.001 |
|  | Adv | .12 | [ .07, .17] | 4.38 | <.001 |
|  | Mat | .09 | [ .03, .14] | 3.22 | .001 |
|  | Pos | -.22 | [-.28, -.16] | -6.93 | <.001 |
|  | Neg | .09 | [ .02, .15] | 2.76 | .006 |
|  | Dec | .03 | [-.03, .08] | 0.96 | .337 |
|  | Soc | .03 | [-.03, .08] | 0.89 | .373 |
|  | age | -.03 | [-.08, .02] | -1.28 | .200 |
|  | gender | .17 | [ .07, .28] | 3.18 | .001 |

*N* = 1,353. Depicted are multiple regression analyses with situation characteristics during
COVID-19 restrictions and age and gender (1 = male) in the prediction of subjective well-being

(SWB) during COVID-19 restrictions and general negative appraisal (GNA) during COVID-19
restrictions. Dut = Duty, Int = Intellect, Adv = Adversity, Mat = Mating, Pos = pOsitivity,
Neg = Negativity, Dec = Deception, Soc = Sociality.
