# Supplementary material for: The situation during the COVID-19 pandemic: A snapshot in Germany
Source: PLoS One. 2021 Feb 12;16(2):e0245719. doi: 10.1371/journal.pone.0245719 (PMC7880467; doi:10.1371/journal.pone.0245719)
Supplement: S5 Table — (DOCX) [file pone.0245719.s005.docx]

**S5 Table. Mediation analyses with age and gender as additional predictors of the mediator and outcome.**

|  | Trait | Total | Indirect effect via | | | | | | | |
| --- | --- | --- | --- | --- | --- | --- | --- | --- | --- | --- |
|  |  |  | Dut | Int | Adv | Mat | Pos | Neg | Dec | Soc |
| SWB during |  |  |  |  |  |  |  |  |  |  |
|  | E | **.19 [.13, .24]** | .01 [.00, .01] | **.05 [.03, .07]** | .02 [.01, .04] | .01 [.00, .03] | **.17 [.13, .20]** | .02 [.00, .04] | .00 [.00, .00] | **.04 [.02, .05]** |
|  | A | **.13 [.07, .18]** | .00 [-.01, .00] | .02 [.01, .04] | **.04 [.02, .06]** | .00 [-.01, .01] | **.11 [.07, .15]** | **.04 [.02, .06]** | .00 [.00, .01] | **.03 [.02, .05]** |
|  | C | **.13 [.07, .18]** | .01 [.00, .02] | .01 [-.01, .03] | **.04 [.03, .06]** | .01 [.00, .02] | **.10 [.06, .14]** | **.04 [.02, .06]** | .00 [.00, .01] | .02 [.01, .03] |
|  | N | **-.50 [-.54, -.45]** | -.01 [-.01, .00] | **-.05 [-.07, -.03]** | **-.04 [-.06, -.03]** | -.01 [-.01, .00] | **-.25 [-.29, -.22]** | **-.09 [-.11, -.07]** | .00 [.00, .00] | **-.02 [-.03, -.01]** |
|  | O | .10 [.04, .16] | .00 [.00, .01] | .11 [.08, .13] | .01 [.00, .03] | .00 [.00, .01] | .09 [.05, .13] | .00 [-.02, .03] | .00 [.00, .00] | .02 [.01, .03] |
|  | H | .08 [.02, .13] | .00 [.00, .01] | .02 [.01, .04] | .02 [.00, .03] | -.01 [-.02, .00] | .05 [.01, .09] | .02 [.00, .04] | .00 [.00, .01] | .01 [.00, .03] |
|  | Narc | -.04 [-.09, .02] | -.01 [-.01, .00] | .04 [.02, .06] | -.02 [-.04, -.01] | .01 [.00, .02] | -.02 [-.06, .02] | -.04 [-.06, -.01] | -.01 [-.01, .00] | .02 [.01, .03] |
|  | Mach | -.04 [-.09, .01] | .00 [-.01, .01] | .02 [.00, .03] | -.03 [-.05, -.02] | .01 [.00, .02] | -.01 [-.05, .03] | -.03 [-.06, -.01] | -.01 [-.03, .01] | .00 [-.01, .01] |
|  | Psyc | -.07 [-.12, -.01] | .00 [-.01, .01] | -.02 [-.04, .00] | -.02 [-.04, -.01] | .01 [.00, .02] | -.06 [-.10, -.03] | -.01 [-.04, .01] | .00 [-.01, .00] | -.03 [-.04, -.02] |
| GNA during |  |  |  |  |  |  |  |  |  |  |
|  | E | .02 [-.04, .08] | .00 [-.01, .00] | -.04 [-.06, -.02] | -.02 [-.03, -.01] | .00 [-.01, .01] | -.08 [-.11, -.06] | -.01 [-.02, .00] | .00 [.00, .00] | -.02 [-.03, -.01] |
|  | A | **-.16 [-.22, -.10]** | .00 [.00, .01] | -.02 [-.03, .00] | **-.03 [-.04, -.02]** | .00 [.00, .00] | **-.05 [-.07, -.03]** | -.02 [-.03, -.01] | .00 [-.01, .00] | -.01 [-.02, .00] |
|  | C | -.06 [-.11, .00] | .00 [-.01, .00] | -.01 [-.02, .00] | -.03 [-.05, -.02] | .00 [.00, .01] | -.05 [-.07, -.03] | -.02 [-.03, -.01] | -.01 [-.01, .00] | -.01 [-.01, .00] |
|  | N | **.12 [.06, .18]** | .00 [.00, .01] | **.05 [.03, .06]** | **.05 [.03, .07]** | .00 [-.01, .00] | **.14 [.11, .18]** | **.06 [.04, .08]** | .00 [.00, .01] | .01 [.00, .02] |
|  | O | -.09 [-.14, -.03] | .00 [-.01, .00] | -.07 [-.10, -.05] | -.01 [-.02, .00] | .00 [.00, .00] | -.04 [-.06, -.02] | .00 [-.01, .01] | .00 [.00, .00] | -.01 [-.01, .00] |
|  | H | **-.12 [-.18, -.05]** | .00 [-.01, .00] | -.02 [-.03, .00] | -.01 [-.03, .00] | .00 [-.01, .00] | -.02 [-.04, -.01] | -.01 [-.02, .00] | .00 [-.01, .00] | .00 [-.01, .00] |
|  | Narc | .01 [-.05, .07] | .00 [.00, .01] | -.03 [-.04, -.01] | .02 [.01, .03] | .00 [.00, .01] | .01 [-.01, .03] | .02 [.01, .03] | .01 [.00, .02] | -.01 [-.01, .00] |
|  | Mach | .06 [.00, .12] | .00 [.00, .00] | -.01 [-.02, .00] | .03 [.01, .04] | .00 [.00, .01] | .01 [-.01, .02] | .02 [.01, .03] | .01 [.00, .03] | .00 [-.01, .00] |
|  | Psyc | **.19 [.13, .25]** | .00 [.00, .00] | .01 [.00, .02] | .02 [.01, .03] | .00 [.00, .01] | .03 [.01, .05] | .01 [.00, .02] | .00 [.00, .01] | .01 [.00, .02] |

*N* = 1,353. Presented are the simple mediation analyses. One mediation model for each trait – situation characteristic – dependent variable combination was fitted. Total = direct effect in a model without a mediator and without age and gender. Indirect effect via = indirect effect mediated via individual situation characteristics. 95%-CIs are given in parentheses. Direct effects printed in bold are significant at α = .001. Indirect effects are printed in bold if they are significant at α = .001, have the same direction as the total effect, and the total effect is significant at α = .001. Direct effects when controlling for each individual mediator were omitted but can be found at osf.io/buvp2. SWB = subjective well-being, GNA = general negative appraisal. Dut = Duty, Int = Intellect, Adv = Adversity, Mat = Mating, Pos = pOsitivity, Neg = Negativity, Dec = Deception, Soc = Sociality, E = Extraversion, A = Agreeableness, C = Conscientiousness, N = Neuroticism, O = Openness, H = Honesty-Humility, Narc = Narcissism, Mach = Machiavellianism, Psyc = Psychopathy.
