# Supplementary material for: The situation during the COVID-19 pandemic: A snapshot in Germany
Source: PLoS One. 2021 Feb 12;16(2):e0245719. doi: 10.1371/journal.pone.0245719 (PMC7880467; doi:10.1371/journal.pone.0245719)
Supplement: S6 Table — (DOCX) [file pone.0245719.s006.docx]

**S6 Table. Multiple mediation analyses with age and gender as additional predictors of each mediator and the outcome.**

|  | Trait | Total | Direct | Indirect total | Indirect effect via | | | | | | | |
| --- | --- | --- | --- | --- | --- | --- | --- | --- | --- | --- | --- | --- |
|  |  |  |  |  | Dut | Int | Adv | Mat | Pos | Neg | Dec | Soc |
| SWB during | E | **.19 [.13, .24]** | .02 [-.02, .06] | **.17 [.13, .21]** | .00 [.00, .01] | **.02 [.01, .03]** | .01 [.00, .01] | .00 [-.01, .00] | **.13 [.10, .17]** | .01 [.00, .02] | .00 [.00, .00] | .00 [-.01, .00] |
|  | A | **.13 [.07, .18]** | .01 [-.04, .05] | **.13 [.08, .17]** | .00 [-.01, .00] | .01 [.00, .02] | .01 [.01, .02] | .00 [.00, .00] | **.09 [.06, .12]** | .02 [.01, .03] | .00 [-.01, .00] | .00 [-.01, .00] |
|  | C | **.13 [.07, .18]** | .01 [-.03, .05] | **.12 [.08, .16]** | .01 [.00, .01] | .00 [.00, .01] | **.01 [.01, .02]** | .00 [-.01, .00] | **.08 [.05, .11]** | .02 [.01, .03] | .00 [-.01, .00] | .00 [-.01, .00] |
|  | N | **-.50 [-.54, -.45]** | **-.19 [-.24, -.15]** | **-.31 [-.35, -.27]** | .00 [-.01, .00] | **-.02 [-.04, -.01]** | -.02 [-.03, -.01] | .00 [.00, .01] | **-.21 [-.25, -.18]** | **-.06 [-.08, -.04]** | .00 [.00, .00] | .00 [.00, .01] |
|  | O | .10 [.04, .16] | -.02 [-.06, .02] | .12 [.08, .16] | .00 [.00, .01] | .04 [.03, .06] | .00 [.00, .01] | .00 [.00, .00] | .07 [.04, .10] | .00 [-.01, .01] | .00 [.00, .00] | .00 [-.01, .00] |
|  | H | .08 [.02, .13] | .01 [-.03, .05] | .07 [.02, .11] | .00 [.00, .01] | .01 [.00, .02] | .01 [.00, .01] | .00 [.00, .01] | .04 [.01, .07] | .01 [.00, .02] | .00 [-.01, .00] | .00 [-.01, .00] |
|  | Narc | -.04 [-.09, .02] | .00 [-.04, .04] | -.03 [-.07, .01] | .00 [-.01, .00] | .01 [.01, .02] | -.01 [-.01, .00] | .00 [-.01, .00] | -.02 [-.05, .02] | -.02 [-.03, -.01] | .00 [.00, .01] | .00 [-.01, .00] |
|  | Mach | -.04 [-.09, .01] | -.01 [-.05, .03] | -.03 [-.07, .01] | .00 [.00, .00] | .01 [.00, .01] | -.01 [-.02, .00] | .00 [-.01, .00] | -.01 [-.04, .02] | -.02 [-.03, -.01] | .00 [-.01, .02] | .00 [.00, .00] |
|  | Psyc | -.07 [-.12, -.01] | .00 [-.04, .04] | -.07 [-.11, -.03] | .00 [.00, .00] | -.01 [-.01, .00] | -.01 [-.02, .00] | .00 [-.01, .00] | -.05 [-.08, -.02] | -.01 [-.02, .01] | .00 [.00, .01] | .00 [.00, .01] |
| GNA during | E | .02 [-.04, .08] | **.11 [.05, .16]** | -.09 [-.12, -.06] | .00 [.00, .00] | -.03 [-.04, -.02] | -.01 [-.02, .00] | .01 [.00, .02] | -.06 [-.08, -.04] | .00 [-.01, .00] | .00 [.00, .00] | .00 [-.01, .02] |
|  | A | **-.16 [-.22, -.10]** | -.08 [-.14, -.03] | **-.07 [-.09, -.04]** | .00 [.00, .00] | -.01 [-.02, .00] | -.02 [-.03, -.01] | .00 [.00, .01] | **-.04 [-.05, -.02]** | -.01 [-.02, .00] | .00 [-.01, .01] | .01 [.00, .02] |
|  | C | -.06 [-.11, .00] | .02 [-.03, .08] | -.06 [-.09, -.03] | .00 [-.01, .01] | -.01 [-.02, .00] | -.02 [-.03, -.01] | .01 [.00, .02] | -.03 [-.05, -.02] | -.01 [-.02, .00] | .00 [-.01, .00] | .00 [.00, .01] |
|  | N | **.12 [.06, .18]** | -.08 [-.14, -.02] | **.20 [.16, .24]** | .00 [.00, .00] | **.04 [.02, .05]** | **.03 [.02, .05]** | -.01 [-.02, .00] | **.11 [.08, .14]** | .03 [.01, .06] | .00 [.00, .01] | .00 [-.01, .00] |
|  | O | -.09 [-.14, -.03] | -.01 [-.06, .05] | -.08 [-.11, -.06] | .00 [.00, .00] | -.05 [-.08, -.03] | -.01 [-.01, .00] | .00 [.00, .01] | -.03 [-.05, -.02] | .00 [-.01, .00] | .00 [.00, .00] | .00 [.00, .01] |
|  | H | **-.12 [-.18, -.05]** | -.05 [-.10, .01] | **-.05 [-.07, -.02]** | .00 [.00, .00] | -.01 [-.02, .00] | -.01 [-.02, .00] | -.01 [-.02, .00] | -.02 [-.03, .00] | .00 [-.01, .00] | .00 [-.01, .00] | .00 [.00, .01] |
|  | Narc | .01 [-.05, .07] | -.03 [-.09, .02] | .02 [-.01, .05] | .00 [.00, .00] | -.02 [-.03, -.01] | .01 [.00, .02] | .01 [.00, .02] | .01 [-.01, .02] | .01 [.00, .02] | .00 [.00, .01] | .00 [.00, .01] |
|  | Mach | .06 [.00, .12] | .00 [-.05, .06] | .03 [.01, .06] | .00 [.00, .00] | -.01 [-.02, .00] | .01 [.01, .03] | .01 [.00, .02] | .00 [-.01, .02] | .01 [.00, .02] | .01 [-.01, .02] | .00 [.00, .00] |
|  | Psyc | **.19 [.13, .25]** | **.13 [.07, .19]** | .04 [.02, .07] | .00 [.00, .00] | .01 [.00, .02] | .01 [.00, .02] | .01 [.00, .01] | .02 [.01, .04] | .00 [.00, .01] | .00 [-.01, .01] | -.01 [-.02, .00] |

*N* = 1,353. Presented are multiple mediation analyses. One mediation model for each trait – dependent variable combination was fitted. Total = direct effect in a model without mediators and without age and gender. Direct = direct effect remaining in models with all eight mediators, age, and gender included. Indirect effect via = indirect effect mediated via situation characteristics. 95%-CIs are given in parentheses. Direct effects printed in bold are significant at α = .001. Indirect effects are printed in bold if they are significant at α = .001, have the same direction as the total effect, and the total effect is significant at α = .001. More detailed models can be found at osf.io/buvp2. SWB = subjective well-being, GNA = general negative appraisal. Dut = Duty, Int = Intellect, Adv = Adversity, Mat = Mating, Pos = pOsitivity, Neg = Negativity, Dec = Deception, Soc = Sociality, E = Extraversion, A = Agreeableness, C = Conscientiousness, N = Neuroticism, O = Openness, H = Honesty-Humility, Narc = Narcissism, Mach = Machiavellianism, Psyc = Psychopathy.
