# Supplementary material for: The situation during the COVID-19 pandemic: A snapshot in Germany
Source: PLoS One. 2021 Feb 12;16(2):e0245719. doi: 10.1371/journal.pone.0245719 (PMC7880467; doi:10.1371/journal.pone.0245719)
Supplement: S7 Table — (DOCX) [file pone.0245719.s007.docx]

**S7 Table. Robustness analyses with respect to order effects.**

|  | Diff:  During - Before  Order 1 | Diff:  During - Before  Order 2 | Diff:  During (Order 2) -Before (Order 1) | Diff:  During (Order 2) - During (Order 1) | Var Ratio: During/Before  Order 1 | Var Ratio: During/Before  Order 2 | Var_Ratio:  During (Order 2) / Before (Order 1) | Var_Ratio:  During (Order 2) / During (Order 1) |
| --- | --- | --- | --- | --- | --- | --- | --- | --- |
| Dut | *d*_z_ = -0.38 [-0.44, -0.31] | *d*_z_ = -0.46 [-0.52, -0.40] | *d* = -0.39 [-0.50, -0.28] | *d* = -0.05 [-0.16, 0.05] | 1.47 [1.27, 1.72] | 1.86 [1.60, 2.18] | 1.74 [1.45, 2.10] | 1.18 [1.01, 1.39] |
| Int | *d*_z_ = -0.45 [-0.51, -0.38] | *d*_z_ = -0.49 [-0.56, -0.42] | *d* = -0.48 [-0.58, -0.37] | *d* = -0.09 [-0.20, 0.02] | 1.14 [1.04, 1.26] | 1.26 [1.14, 1.39] | 1.26 [1.11, 1.44] | 1.11 [0.98, 1.25] |
| Adv | *d*_z_ = -0.03 [-0.11, 0.04] | *d*_z_ = -0.20 [-0.27, -0.12] | *d* = -0.23 [-0.34, -0.12] | *d* = -0.19 [-0.30, -0.08] | 1.17 [1.06, 1.29] | 1.16 [1.02, 1.31] | 1.15 [0.96, 1.36] | 0.98 [0.83, 1.17] |
| Mat | *d*_z_ = -0.34 [-0.41, -0.27] | *d*_z_ = -0.48 [-0.55, -0.41] | *d* = -0.42 [-0.53, -0.31] | *d* = -0.12 [-0.23, -0.01] | 0.90 [0.80, 1.00] | 0.93 [0.84, 1.03] | 0.90 [0.78, 1.02] | 1.00 [0.87, 1.15] |
| Pos | *d*_z_ = -0.42 [-0.49, -0.36] | *d*_z_ = -0.50 [-0.57, -0.43] | *d* = -0.54 [-0.65, -0.44] | *d* = -0.10 [-0.21, 0.00] | 1.32 [1.17, 1.49] | 1.52 [1.34, 1.72] | 1.51 [1.29, 1.76] | 1.14 [0.98, 1.32] |
| Neg | *d*_z_ = -0.18 [-0.26, -0.11] | *d*_z_ = -0.11 [-0.19, -0.04] | *d* = -0.16 [-0.27, -0.05] | *d* = 0.02 [-0.09, 0.13] | 1.18 [1.07, 1.31] | 1.20 [1.07, 1.33] | 1.27 [1.11, 1.45] | 1.07 [0.94, 1.22] |
| Dec | *d*_z_ = -0.07 [-0.15, 0.00] | *d*_z_ = -0.12 [-0.20, -0.05] | *d* = -0.18 [-0.28, -0.07] | *d* = -0.12 [-0.23, -0.02] | 0.95 [0.87, 1.05] | 1.03 [0.92, 1.15] | 0.98 [0.83, 1.13] | 1.03 [0.88, 1.20] |
| Soc | *d*_z_ = -0.40 [-0.46, -0.34] | *d*_z_ = -0.48 [-0.56, -0.41] | *d* = -0.51 [-0.61, -0.40] | *d* = -0.12 [-0.23, -0.02] | 1.40 [1.26, 1.58] | 1.58 [1.39, 1.80] | 1.60 [1.38, 1.87] | 1.14 [1.00, 1.30] |
| SWB | *d*_z_ = -0.60 [-0.68, -0.53] | *d*_z_ = -0.65 [-0.72, -0.57] | *d* = -0.79 [-0.91, -0.68] | *d* = -0.08 [-0.19, 0.03] | 1.53 [1.33, 1.76] | 1.46 [1.28, 1.68] | 1.43 [1.21, 1.70] | 0.93 [0.82, 1.07] |

*N* = 1,353. Presented are mean level differences (Diff) and variance ratios (Var_Ratio). Order 1 = before followed by during, Order 2 = during followed by before. During (Order 2) vs. Before (Order 1) represents the most conservative comparison (between subjects) since it only compares items that are presented first and are hence less affected by contrast effects. Shown are effect sizes with bootstrapped 95%-CIs. *d_z_* = within-subjects standardized mean difference, *d* = Cohen’s *d*. SWB = subjective well-being, Dut = Duty, Int = Intellect, Adv = Adversity, Mat = Mating, Pos = pOsitivity, Neg = Negativity, Dec = Deception, Soc = Sociality.
